# Analysis of renewable energy resources based on frank power aggregation operators and EDAS method for circular bipolar complex fuzzy uncertainty

**DOI:** 10.1016/j.heliyon.2024.e37872

**Published:** 2024-09-14

**Authors:** Zeeshan Ali, Khumara Ashraf, Khizar Hayat

**Affiliations:** a Department of Information Management, National Yunlin University of Science and Technology, 123 University Road, Section 3, Douliou, Yunlin, 64002, Taiwan; b Department of Mathematics and Statistics, Riphah International University Islamabad, 44000, Pakistan; c Department of Mathematics, University of Kotli, AJ&K, Pakistan

**Keywords:** Circular bipolar complex fuzzy sets, EDAS techniques, Frank power aggregation operators, Renewable energy resources, Decision-making problems

## Abstract

The model of circular bipolar complex fuzzy (Cir-BCF) sets computed based on the membership function, non-membership function, and radius among both functions for each value of the universal set. The technique of the Cir-BCF set is the modified or extended form of fuzzy sets, complex fuzzy sets, bipolar fuzzy sets, bipolar complex fuzzy sets, and simple circular bipolar fuzzy sets to cope with uncertain and vague information. In this manuscript, we describe the novel technique of frank operational laws based on Cir-BCF values for frank t-norm and frank t-conorm. Further, we simplify the model of Cir-BCF frank power averaging (Cir-BCFFPA), Cir-BCF frank power weighted averaging (Cir-BCFFPWA), Cir-BCF frank power geometric (Cir-BCFFPG), Cir-BCF frank power weighted geometric (Cir-BCFFPWG) operators, and highlighted their valuable properties, called idempotency, monotonicity, and boundedness. Moreover, analysis of renewable energy resources is very awkward and complicated, but it is natural sources of energy that are refilled constantly or relativeness quickly on a human timescale. Further, solar energy, hydroelectric energy, geothermal energy, biomass energy, and wind energy are five major sources of energy, but it is complex to find which one is the best and which one is worst. For evaluating the above problems, we construct the technique of evaluation based on the distance from the average solution (EDAS) method under the presence of the Cir-BCF numbers (Cir-BCFNs). At the end, we arrange the comparison between proposed and existing techniques based on some numerical examples to describe the validity and proficiency of the initiated information.

## 1 Introduction

The application of renewable energy resources [1] is widely discussed and utilized in many fields, because of their features, where renewable energy resources are sources of energy that are filled naturally and can be employed without reducing their reserves. Further, the application of renewable energy resources is very common and reliable for evaluating the solution of complex and ambiguous kinds of problems under the consideration of the decision-making process [2]. The technique of the MADM problem is part of the decision-making problem, which is used for finding the best decision among the collection of alternatives under the consideration of the crisp set theory. Moreover, the range of the crisp set is very narrow, because we have just zero and one but not partial value and due to this reason, much information was lost by experts during the decision-making procedure under the presence of the crisp set theory. For this, Zadeh [3] initiated the theory of fuzzy set (FS) theory, where the grade of membership function in FSs is defined from any fixed set to unit interval. The concept of FS is very reliable because of positive membership grades, but it is not enough because of negative membership grades, where the information of positive and negative membership grades is part of many genuine life problems. Therefore, Zhang [4] presented the technique of bipolar fuzzy set (BFS) in 1994, where the BFSs were computed with two same grades but different directions, where the range of positive membership grade is [0, 1] and the range of the negative membership grade is [−1, 0]. The technique of complex fuzzy set (CFS) [5] is very reliable when a decision-maker faces two-dimensional information in some genuine life problems. The technique of membership grade in CFSs is computed in the shape of complex numbers, where the real and imaginary parts are dealt with two-dimensional information. Further, the technique of FS is the part of CFS, when we consider the imaginary part will be equal to zero. The theory of FSs, BFSs, and CFSs has received various attention from different scholars, but during the decision-making procedure, experts have faced a problem, when an expert provides information on the theory of positive and negative membership values in the shape of complex numbers, then decision-makers have failed to deal with it. For this, the technique, or the theory of bipolar complex fuzzy set (BCFS) was initiated by Mahmood and Rehman [6] by including the grade of positive and negative functions in the shape of complex numbers, where, ${{}^{\circ}C}_{\kappa_{B}}^{P}\left(Z\right)={{}^{\circ}C}_{\kappa_{B-1}}^{RP}\left(Z\right)+℩{{}^{\circ}C}_{\kappa_{B-1}}^{℩P}\left(Z\right)$ and ${{}^{\circ}C}_{\kappa_{B}}^{N}\left(Z\right)={{}^{\circ}C}_{\kappa_{B-1}}^{RN}\left(Z\right)+℩{{}^{\circ}C}_{\kappa_{B-1}}^{℩N}\left(Z\right)$ suggests the positive and negative membership functions.

### 1.1 Literature review

The mathematical model of FS is very common but flexible and reliable for depicting uncertain and vague information. Because of their structure, the technique of FSs has been utilized by many individuals. Some well-known extensions are described in the form, for instance, the model of fuzzy superior Mandelbrot set was invented by Mahmood and Ali [7]. The model of generalized fuzzy superior Mandelbrot set was discovered by Ince and Ersoy [8]. Moreover, the model of FS is very reliable but not good for evaluating some genuine-life problems, where from the above analysis is it clear that the technique of BFS is very flexible because of their positive and negative membership function. The technique of BFSs has received much attention in different fields, for instance, Hamacher operators for BFS was initiated by Wei et al. [9], robust aggregation operators for BFS were derived by Jana et al. [10], similarity measures for BFS were presented by Riaz et al. [11], decision-making problems for BFS was exposed by Alghamdi et al. [12], TOPSIS technique for BFS was invented by Akram et al. [13], and VIKOR technique for BFS was presented by Riaz and Tehrim [14]. After the construction of FS, the technique of CFS has received a lot of attention from different scholars. Some applications of CFSs are described in the following shape, for instance, distance measures for CFSs were derived by Liu et al. [15] and neighborhood operators for CFSs were derived by Mahmood et al. [16]. Further, some dominant applications of BCFS are computed in the shape of bipolar complex fuzzy credulity aggregation operators [17] and bipolar complex fuzzy linear systems [18]. The positive truth grade is not enough for managing awkward and vague kind of problems, but in the consideration of the positive truth grade and radius function, it is much better than existing FSs, therefore, Tusor and Varkonyi-Koczy [19] presented the novel technique of circular fuzzy set (Cir-FSs), where the truth function and radius function are the major parts of the Cir-FSs. Further, Han et al. [20] developed some fuzzy measures under the consideration of the Cir-FSs. Additionally, Keshavarz Ghorabaee et al. [21] presented the technique of the EDAS method for classical set theory. In 1997, Klement et al. [22] wrote a book titled triangular norm, which contained the mathematical shape of all existing norms, where frank t-norm (FTN) and frank t-conorm (FTCN) are one of them, proposed by Frank [23]. Furthermore, operators based on FSs were initiated by Yager [24]. Further, Bi et al. [25] exposed the technique of arithmetic operators for CFSs. In 2018, the technique of geometric operators for CFSs was proposed by Bi et al. [26]. Additionally, Hu et al. [27] presented the power operators for CFSs. Moreover, Mahmood et al. [28] identified the classification of the aggregation operators for BCFSs and their applications. In 2021, The technique of Hamacher operators for BCFSs was initiated by Mahmood et al. [29]. Further, frank operators for BCFSs were initiated by Naeem et al. [30].

### 1.2 Research gap and major problems

In the above paragraph, we briefly discussed different kinds of techniques, methods, and measures based on the model of FSs and their extensions. From the above analysis, we concluded or finalized that the following problems are faced by experts during the decision-making procedure and because of these reasons they lost a lot of data, such as

1) How to develop new operational laws based on some existing norms?
2) How to aggregate distinct criteria values and arrange the overall preference values?
3) How to arrange the ranking values for exposing the best or excellent alternative among the collection of alternatives?

Hence, the purpose of this study is to provide a new decision-making technique for addressing the problems of the MADM model under the consideration of the Cir-BCF set with frank aggregation operators. The model of Aczel-Alsina information based on the Cir-BCF set (Cir-BCFS) was presented by Ali et al. [31]. The model of Cir-BCFS is very dominant because of their structure, where the technique of BFS is the special case of the Cir-BCFS. It is represented by a circle with a radius of each element consisting of degrees of membership and non-membership functions. Further, the technique of frank aggregation operators for extended fuzzy sets was initiated by Seikh and Mandal [32]. The technique of frank operators for picture fuzzy sets was presented by Seikh and Mandal [33]. In 2023, Seikh and Mandal [34] evaluated the Archimedean operators for extended fuzzy sets. Some special cases of the Cir-BCFS are described in Table 1.

**Table 1:** Extension of the FSs to Cir-BCFSs.

Methods	Membership function	Non-membership function	Radius function	Complex-valued information
FS	Yes	no	No	No
BFS	Yes	Yes	No	No
CFS	Yes	No	No	No
BCFS	Yes	Yes	No	No
Circular FS	Yes	no	Yes	No
Circular CFS	Yes	no	Yes	Yes
Circular BFS	Yes	yes	Yes	No
Cir-BCFS	Yes	yes	Yes	Yes

From the information in Table 1, we can see that the technique of Cir-BCFS is very reliable and dominant because of its features. Further, the technique of Cir-BCFS was initiated in 2024 which is novel and beneficial. Further, only Aczel-Alsina operators were proposed based on Cir-BCFSs. The major question is whether the Aczel-Alsina operators are enough for aggregating the collection of information into a singleton set. Why do we not propose the technique of frank aggregation operators which is modified and superior to Aczel-Alsina operators? Further, it is also very important to develop the technique of EDAS method based on Cir-BCFS which has not been analyzed yet by anyone. Because of these problems, we aim to evaluate the technique of frank operators and, the EDAS method, and describe their application in renewable energy resources.

### 1.3 Motivation/advantages/major contribution of the proposed theory

**Fig. 1:**
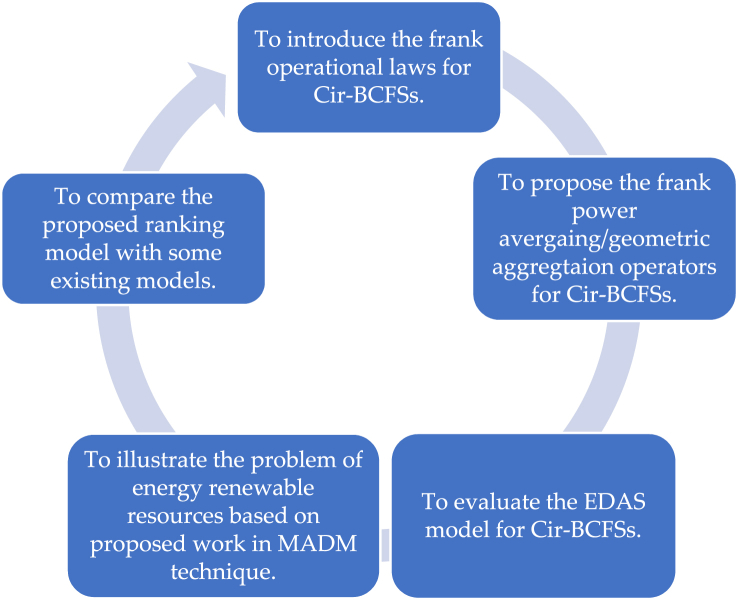
Geometrical abstract of the proposed theory.

Aggregating the collection of information into a singleton set is very complex, especially in the presence of the Cir-BCF values, because to date only the technique of Aczel-Alsina (AA) operators was proposed. However, we aim to develop more operators based on different norms and also compare their ranking values to describe the stability and supremacy of the proposed theory. The major contribution of the proposed theory is listed below:

1) To describe the novel technique of frank operational laws based on Cir-BCF values for frank t-norm and frank t-conorm.
2) To simplify the model of Cir-BCFFPA, Cir-BCFFPWA, Cir-BCFFPG, and Cir-BCFFPWG operators, and highlighted their valuable properties, called idempotency, monotonicity, and boundedness.
3) To construct the technique of EDAS method under the presence of the Cir-BCFNs.
4) To arrange the comparison between proposed and existing techniques based on some numerical examples to describe the validity and proficiency of the initiated information. The geometrical representation of the proposed theory is listed in the form of Fig. 1.

From our brief discussion, it is obvious that the proposed techniques are very capable and reliable in coping with the raised questions which are discussed in subsection 1.2. The major advantages of the presented theory are listed below:

1) The model of power, averaging, and geometric (algebraic, frank, AA) operators for FSs.
2) The model of power, averaging, and geometric (algebraic, frank, AA) operators for CFSs.
3) The model of power, averaging, and geometric (algebraic, frank, AA) operators for BFSs.
4) The model of power, averaging, and geometric (algebraic, frank, AA) operators for BCFSs.
5) The model of power, averaging, and geometric (algebraic, frank, AA) operators for circular FSs.
6) The model of power, averaging, and geometric (algebraic, frank, AA) operators for circular CFSs.
7) The model of power, averaging, and geometric (algebraic, frank, AA) operators for circular BFSs.

These all are the special cases of the invented theory. Under the consideration of the above advantages and based on the effectiveness and supremacy of the Cir-BCFS, we aim to evaluate the technique of the EDAS method, frank operators, and also evaluate the problem of energy renewable resources based on Cir-BCFS is very reliable and beneficial for genuine-life problems. Based on the above analysis, the summary of the proposed manuscript is listed below:

1) In Section 2, we discussed the old technique of BCFSs, and their related laws based on universal set η. Further, we described the technique of Frank's operational laws and power operators for any collection of non-negative information.
2) In Section 3, we analyzed some operational laws based on algebraic and frank norms for Cir-BCFSs.
3) In Section 4, we presented the technique of Cir-BCFFPA, Cir-BCFFPWA, Cir-BCFFPG, and Cir-BCFFPWG operators. Some properties for the above operators are also derived.
4) In Section 5, we constructed the technique of the EDAS method under the presence of the Cir-BCFNs.
5) In Section 6, for resolving the problem of renewable energy resources, the technique of the MADM method is computed for the proposed operators.
6) In Section 7, we arranged the comparison between proposed and existing techniques to enhance the worth of the initiated information. Some final remarks are part of Section 8.

## 2 Preliminaries

This section describes the old idea of BCFSs, Cir-BCFSs, and reliable laws with the basic idea of Frank's operational laws and power operators for any collection of non-negative information.

Definition 1 [6] Assume a set η, a set $\kappa_{B}$ over η is known as BCFS and deduced as

$$=\left\{\left(Z,\left({{}^{\circ}C}_{\kappa_{B-1}}^{RP}\left(Z\right)+℩{{}^{\circ}C}_{\kappa_{B-1}}^{℩P}\left(Z\right),{{}^{\circ}C}_{\kappa_{B-1}}^{RN}\left(Z\right)+℩{{}^{\circ}C}_{\kappa_{B-1}}^{℩N}\left(Z\right)\right)\right)|Z\in \eta \right\}$$

where, ${{}^{\circ}C}_{\kappa_{B}}^{P}\left(Z\right)={{}^{\circ}C}_{\kappa_{B-1}}^{RP}\left(Z\right)+℩{{}^{\circ}C}_{\kappa_{B-1}}^{℩P}\left(Z\right),{{}^{\circ}C}_{\kappa_{B}}^{N}\left(Z\right)={{}^{\circ}C}_{\kappa_{B-1}}^{RN}\left(Z\right)+℩{{}^{\circ}C}_{\kappa_{B-1}}^{℩N}\left(Z\right)$ suggests the positive and negative membership functions with a simple shape of BCFN, such as $\kappa_{B}=\left({{}^{\circ}C}_{\kappa_{B}}^{P},{{}^{\circ}C}_{\kappa_{B}}^{N}\right)=\left({{}^{\circ}C}_{\kappa_{B}}^{RP}+℩{{}^{\circ}C}_{\kappa_{B}}^{℩P},{{}^{\circ}C}_{\kappa_{B}}^{RN}+℩{{}^{\circ}C}_{\kappa_{B}}^{℩N}\right)$.

Definition 2 [30] Assume that $\kappa_{B-1}=\left({{}^{\circ}C}_{\kappa_{B-1}}^{P},{{}^{\circ}C}_{\kappa_{B-1}}^{N}\right)=\left({{}^{\circ}C}_{\kappa_{B-1}}^{RP}+℩{{}^{\circ}C}_{\kappa_{B-1}}^{℩P},{{}^{\circ}C}_{\kappa_{B-1}}^{RN}+℩{{}^{\circ}C}_{\kappa_{B-1}}^{℩N}\right)$ and $\kappa_{B-2}=\left({{}^{\circ}C}_{\kappa_{B-2}}^{P},{{}^{\circ}C}_{\kappa_{B-2}}^{N}\right)=\left({{}^{\circ}C}_{\kappa_{B-2}}^{RP}+℩{{}^{\circ}C}_{\kappa_{B-2}}^{℩P},{{}^{\circ}C}_{\kappa_{B-2}}^{RN}+℩{{}^{\circ}C}_{\kappa_{B-2}}^{℩N}\right)$, are two BCFNs ϜℲ>1, and ∄>O with and then Frank operational laws for BCFNs, such as

$$\kappa_{B-1}⊕\kappa_{B-2}=\left(\begin{array}{c} 1-\log_{ϜℲ}\left(1+\frac{\left({ϜℲ}^{1-{{}^{\circ}C}_{\kappa_{B-1}}^{RP}}-1\right)\left({ϜℲ}^{1-{{}^{\circ}C}_{\kappa_{B-2}}^{RP}}-1\right)}{ϜℲ-1}\right)\\ +℩\left(1-\log_{ϜℲ}\left(1+\frac{\left({ϜℲ}^{1-{{}^{\circ}C}_{\kappa_{B-1}}^{℩P}}-1\right)\left({ϜℲ}^{1-{{}^{\circ}C}_{\kappa_{B-2}}^{℩P}}-1\right)}{ϜℲ-1}\right)\right),\\ -\log_{ϜℲ}\left(1+\frac{\left({ϜℲ}^{-{{}^{\circ}C}_{\kappa_{B-1}}^{RN}}-1\right)\left({ϜℲ}^{-{{}^{\circ}C}_{\kappa_{B-2}}^{RN}}-1\right)}{ϜℲ-1}\right)\\ +℩\left(-\left(\log_{ϜℲ}\left(1+\frac{\left({ϜℲ}^{-{{}^{\circ}C}_{\kappa_{B-1}}^{℩N}}-1\right)\left({ϜℲ}^{-{{}^{\circ}C}_{\kappa_{B-2}}^{℩N}}-1\right)}{ϜℲ-1}\right)\right)\right) \end{array}\right)$$

$$\kappa_{B-1}⊗\kappa_{B-2}=\left(\begin{array}{c} {\;\log}_{ϜℲ}\left(1+\frac{\left({ϜℲ}^{{{}^{\circ}C}_{\kappa_{B-1}}^{RP}}-1\right)\left({ϜℲ}^{{{}^{\circ}C}_{\kappa_{B-2}}^{RP}}-1\right)}{ϜℲ-1}\right)\\ +℩\left(\log_{ϜℲ}\left(1+\frac{\left({ϜℲ}^{{{}^{\circ}C}_{\kappa_{B-1}}^{℩P}}-1\right)\left({ϜℲ}^{{{}^{\circ}C}_{\kappa_{B-2}}^{℩P}}-1\right)}{ϜℲ-1}\right)\right),\\ -1+\log_{ϜℲ}\left(1+\frac{\left({ϜℲ}^{1+{{}^{\circ}C}_{\kappa_{B-1}}^{RP}}-1\right)\left({ϜℲ}^{1+{{}^{\circ}C}_{\kappa_{B-2}}^{RP}}-1\right)}{ϜℲ-1}\right)\\ +℩\left(-1+\log_{ϜℲ}\left(1+\frac{\left({ϜℲ}^{1+{{}^{\circ}C}_{\kappa_{B-1}}^{RP}}-1\right)\left({ϜℲ}^{1+{{}^{\circ}C}_{\kappa_{B-2}}^{RP}}-1\right)}{ϜℲ-1}\right)\right) \end{array}\right)$$

$$∄\kappa_{B-1}=\left(\begin{array}{c} 1-{\;\log}_{ϜℲ}\left(1+\frac{{\left({ϜℲ}^{1-{{}^{\circ}C}_{\kappa_{B-1}}^{RP}}-1\right)}^{∄}}{{\left(ϜℲ-1\right)}^{∄-1}}\right)\\ +℩\left(1-\log_{ϜℲ}\left(1+\frac{{\left({ϜℲ}^{1-{{}^{\circ}C}_{\kappa_{B-1}}^{℩P}}-1\right)}^{∄}}{{\left(ϜℲ-1\right)}^{∄-1}}\right)\right),\\ -\left({\;\log}_{ϜℲ}\left(1+\frac{{\left({ϜℲ}^{-{{}^{\circ}C}_{\kappa_{B-1}}^{RP}}-1\right)}^{∄}}{{\left(ϜℲ-1\right)}^{∄-1}}\right)\right)\\ +℩\left(-\left({\;\log}_{ϜℲ}\left(1+\frac{{\left({ϜℲ}^{-{{}^{\circ}C}_{\kappa_{B-1}}^{RP}}-1\right)}^{∄}}{{\left(ϜℲ-1\right)}^{∄-1}}\right)\right)\right). \end{array}\right)$$

$${\kappa_{B-1}}^{∄}=\left(\begin{array}{c} {\;\log}_{ϜℲ}\left(1+\frac{{\left({ϜℲ}^{{{}^{\circ}C}_{\kappa_{B-1}}^{RP}}-1\right)}^{∄}}{{\left(ϜℲ-1\right)}^{∄-1}}\right)\\ +℩\left(\log_{ϜℲ}\left(1+\frac{{\left({ϜℲ}^{{{}^{\circ}C}_{\kappa_{B-1}}^{℩P}}-1\right)}^{∄}}{{\left(ϜℲ-1\right)}^{∄-1}}\right)\right),\\ -1+\left({\;\log}_{ϜℲ}\left(1+\frac{{\left({ϜℲ}^{1+{{}^{\circ}C}_{\kappa_{B-1}}^{RP}}-1\right)}^{∄}}{{\left(ϜℲ-1\right)}^{∄-1}}\right)\right)\\ +℩\left({-1+\log}_{ϜℲ}\left(1+\frac{{\left({ϜℲ}^{1+{{}^{\circ}C}_{\kappa_{B-1}}^{RP}}-1\right)}^{∄}}{{\left(ϜℲ-1\right)}^{∄-1}}\right),\right) \end{array}\right)$$

Definition 3 [27] Assume positive numbers as a set, then $PA\left(\kappa_{CB-1},\kappa_{B-2},\kappa_{B-3},\ldots ,\kappa_{B-\ddot{u}}\right)={⊕}_{\varepsilon =1}^{u}\frac{\left(1+T\left(\kappa_{B-\varepsilon }\right)\right)}{\sum_{\varepsilon -1}^{.\ddot{u}}\left(1+T\left(\kappa_{B-\varepsilon }\right)\right)}\kappa_{B-\varepsilon }$ is deduced as power AO. Now $T\left(\kappa_{B-\varepsilon }\right)$ = $\sum_{\begin{array}{c} \varepsilon -1\\ \varepsilon \neq \varepsilon \end{array}}^{.\ddot{u}}\mathrm{Sup}\left(\kappa_{B-\varepsilon },\kappa_{B-\varepsilon }\right)$ and $\mathrm{Sup}\left(\kappa_{B-\varepsilon },\kappa_{B-\varepsilon }\right)$ connotes the support among $\kappa_{B-\varepsilon }$ and $\kappa_{B-\varepsilon }$ along with underneath axioms

$$\mathrm{Sup}\left(\kappa_{B-\varepsilon },\kappa_{B-\varepsilon }\right)\in \left[O,1\right]$$

$$\mathrm{Sup}\left(\kappa_{B-\varepsilon },\kappa_{B-\varepsilon }\right)=\mathrm{Sup}\left(\kappa_{B-\varepsilon },\kappa_{B-\varepsilon }\right)$$

$$\mathrm{Sup}\left(\kappa_{CB-\varepsilon },\kappa_{CB-\varepsilon }\right)\geq \mathrm{Sup}\left(\kappa_{CB-\varepsilon },\kappa_{CB-\varepsilon }\right)\;if\;O\left(\kappa_{CB-\varepsilon },\kappa_{CB-\varphi }\right)<O\left(\kappa_{CB-r},\kappa_{CB-\varepsilon }\right)$$

where O is any distance measure. Further, we discussed the technique of Cir-BCFSs and their operational laws.

Definition 4 [31] Assume a set η, a set $\kappa_{B}$ over η is known as Cir-BCFS and deduced as

$$\kappa_{CB}=\left\{\left(Z,\left({{}^{\circ}C}_{\kappa_{CB}}^{P}\left(Z\right),{{}^{\circ}C}_{\kappa_{CB}}^{N}\left(Z\right),\xi_{\kappa_{CB}}^{P}\left(Z\right)\right)\right)|Z\in \eta \right\}$$

$$=\left\{\left(Z,\left({{}^{\circ}C}_{\kappa_{CB}}^{RP}\left(Z\right)+℩{{}^{\circ}C}_{\kappa_{CB}}^{℩P}\left(Z\right),{{}^{\circ}C}_{\kappa_{CB}}^{RN}\left(Z\right)+℩{{}^{\circ}C}_{\kappa_{CB}}^{℩N}\left(Z\right),\xi_{\kappa_{CB}}^{RP}\left(Z\right)+℩\xi_{\kappa_{CB}}^{℩P}\left(Z\right)\right)\right)|Z\in \eta \right\}$$

where ${{}^{\circ}C}_{\kappa_{CB}}^{P}\left(Z\right)$, ${{}^{\circ}C}_{\kappa_{CB}}^{N}\left(Z\right)$ suggests a positive and negative membership function, and $\xi_{\kappa_{CB}}^{P}\left(Z\right)$ suggests the radius degree with a simple shape of Cir-BCF numbers (Cir-BCFNs), such as $\kappa_{CB}=\left({{}^{\circ}C}_{\kappa_{CB}}^{P},{{}^{\circ}C}_{\kappa_{CB}}^{N},\xi_{\kappa_{CB}}^{P}\right)=\left({{}^{\circ}C}_{\kappa_{CB}}^{RP}+℩{{}^{\circ}C}_{\kappa_{CB}}^{℩P},{{}^{\circ}C}_{\kappa_{CB}}^{RN}+℩{{}^{\circ}C}_{\kappa_{CB}}^{℩N},\xi_{\kappa_{CB}}^{RP}+℩\;\xi_{\kappa_{CB}}^{℩P}\right)$.

Definition 5 [31] Assume that $\kappa_{CB-1}=\left({{}^{\circ}C}_{\kappa_{CB-1}}^{P},{{}^{\circ}C}_{\kappa_{CB-1}}^{N},\xi_{\kappa_{CB-1}}^{P}\right)=\left({{}^{\circ}C}_{\kappa_{CB-1}}^{RP}+℩{{}^{\circ}C}_{\kappa_{CB-1}}^{℩P},{{}^{\circ}C}_{\kappa_{CB-1}}^{RN}+℩{{}^{\circ}C}_{\kappa_{CB-1}}^{℩N},\xi_{\kappa_{CB-1}}^{RP}+℩\;\xi_{\kappa_{CB-1}}^{℩P}\right)$ and $\kappa_{CB-2}=\left({{}^{\circ}C}_{\kappa_{CB-2}}^{P},{{}^{\circ}C}_{\kappa_{CB-2}}^{N},\xi_{\kappa_{CB-2}}^{P}\right)=\left({{}^{\circ}C}_{\kappa_{CB-2}}^{RP}+℩{{}^{\circ}C}_{\kappa_{CB-2}}^{℩P},{{}^{\circ}C}_{\kappa_{CB-2}}^{RN}+℩{{}^{\circ}C}_{\kappa_{CB-2}}^{℩N},\xi_{\kappa_{CB-2}}^{RP}+℩\;\xi_{\kappa_{CB-2}}^{℩P}\right)$ are two Cir-BCFNs with ∄>O, then

$$\kappa_{CB-1}{⊕}_{tc}\kappa_{CB-2}=\left(\begin{array}{c} {{}^{\circ}C}_{\kappa_{CB-1}}^{RP}+{{}^{\circ}C}_{\kappa_{CB-2}}^{RP}-{{}^{\circ}C}_{\kappa_{CB-1}}^{RP}{{}^{\circ}C}_{\kappa_{CB-2}}^{RP}\\ +℩\left({{}^{\circ}C}_{\kappa_{CB-1}}^{℩P}+{{}^{\circ}C}_{\kappa_{CB-2}}^{℩P}-{{}^{\circ}C}_{\kappa_{CB-1}}^{℩P}{{}^{\circ}C}_{\kappa_{CSB-2}}^{℩P}\right),\\ -\left({{}^{\circ}C}_{\kappa_{CB-1}}^{RN}{{}^{\circ}C}_{\kappa_{CB-2}}^{RN}\right)+℩\left(-\left({{}^{\circ}C}_{\kappa_{CB-1}}^{℩N}{{}^{\circ}C}_{\kappa_{CSB-2}}^{℩N}\right)\right),\\ \xi_{\kappa_{CB-1}}^{RP}+\xi_{\kappa_{CB-2}}^{RP}-\xi_{\kappa_{CB-1}}^{RP}\xi_{\kappa_{CSB-2}}^{RP}\\ +℩\left(\xi_{\kappa_{CB-1}}^{℩P}+\xi_{\kappa_{CB-2}}^{℩P}-\xi_{\kappa_{CB-1}}^{℩P}\xi_{\kappa_{CSB-2}}^{℩P}\right) \end{array}\right)$$

$$\kappa_{CB-1}{⊕}_{t}\kappa_{CB-2}=\left(\begin{array}{c} {{}^{\circ}C}_{\kappa_{CB-1}}^{RP}+{{}^{\circ}C}_{\kappa_{CB-2}}^{RP}-{{}^{\circ}C}_{\kappa_{CB-1}}^{RP}{{}^{\circ}C}_{\kappa_{CB-2}}^{RP}\\ +℩\left({{}^{\circ}C}_{\kappa_{CB-1}}^{℩P}+{{}^{\circ}C}_{\kappa_{CB-2}}^{℩P}-{{}^{\circ}C}_{\kappa_{CB-1}}^{℩P}{{}^{\circ}C}_{\kappa_{CB-2}}^{℩P}\right),\\ -\left({{}^{\circ}C}_{\kappa_{CB-1}}^{RN}{{}^{\circ}C}_{\kappa_{CB-2}}^{RN}\right)+℩\left(-\left({{}^{\circ}C}_{\kappa_{CB-1}}^{℩N}{{}^{\circ}C}_{\kappa_{CB-2}}^{℩N}\right)\right),\\ \left(\xi_{\kappa_{CB-1}}^{RP}\;\xi_{\kappa_{CB-2}}^{RP}\right)+℩\left(\xi_{\kappa_{CB-1}}^{℩P}\;\xi_{\kappa_{CB-2}}^{℩P}\right) \end{array}\right)$$

$$\kappa_{CB-1}{⊗}_{tc}\kappa_{CB-2}=\left(\begin{array}{c} {{}^{\circ}C}_{\kappa_{CB-1}}^{RP}{{}^{\circ}C}_{\kappa_{CB-2}}^{RP}+℩{{}^{\circ}C}_{\kappa_{CB-1}}^{℩P}{{}^{\circ}C}_{\kappa_{CB-2}}^{℩P},\\ {{}^{\circ}C}_{\kappa_{CB-1}}^{RN}+{{}^{\circ}C}_{\kappa_{CB-2}}^{RN}+{{}^{\circ}C}_{\kappa_{CB-1}}^{RN}{{}^{\circ}C}_{\kappa_{CB-2}}^{RN}\\ +℩\left({{}^{\circ}C}_{\kappa_{CB-1}}^{℩N}+{{}^{\circ}C}_{\kappa_{CB-2}}^{℩N}+{{}^{\circ}C}_{\kappa_{CB-1}}^{℩N}{{}^{\circ}C}_{\kappa_{CB-2}}^{℩N}\right),\\ \left(\xi_{\kappa_{CB-1}}^{RP}\;\xi_{\kappa_{CB-2}}^{RP}\right)+℩\left(\xi_{\kappa_{CB-1}}^{℩P}\;\xi_{\kappa_{CB-2}}^{℩P}\right) \end{array}\right)$$

$$\kappa_{CB-1}{⊗}_{t}\kappa_{CB-2}=\left(\begin{array}{c} {{}^{\circ}C}_{\kappa_{CB-1}}^{RP}{{}^{\circ}C}_{\kappa_{CB-2}}^{RP}+℩{{}^{\circ}C}_{\kappa_{CB-1}}^{℩P}{{}^{\circ}C}_{\kappa_{CB-2}}^{℩P},\\ {{}^{\circ}C}_{\kappa_{CB-1}}^{RN}+{{}^{\circ}C}_{\kappa_{CB-2}}^{RN}+{{}^{\circ}C}_{\kappa_{CB-1}}^{RN}{{}^{\circ}C}_{\kappa_{CB-2}}^{RN}\\ +℩\left({{}^{\circ}C}_{\kappa_{CB-1}}^{℩N}+{{}^{\circ}C}_{\kappa_{CB-2}}^{℩N}+{{}^{\circ}C}_{\kappa_{CB-1}}^{℩N}{{}^{\circ}C}_{\kappa_{CB-2}}^{℩N}\right),\\ \xi_{\kappa_{CB-1}}^{RP}\;{+\xi }_{\kappa_{CB-2}}^{RP}-\xi_{\kappa_{CB-1}}^{RP}\;\xi_{\kappa_{CB-2}}^{RP}\\ +℩\left(\xi_{\kappa_{CB-1}}^{RP}\;{+\xi }_{\kappa_{CB-2}}^{RP}-\xi_{\kappa_{CB-1}}^{℩P}\;\xi_{\kappa_{CB-2}}^{℩P}\right) \end{array}\right)$$

$${{∄\kappa }_{CB-1}}_{tc}=\left(\begin{array}{c} 1-{\left(1-{{}^{\circ}C}_{\kappa_{CB-1}}^{RP}\right)}^{∄}+℩\left(1-{\left(1-{{}^{\circ}C}_{\kappa_{CB-1}}^{℩P}\right)}^{∄}\right),\\ -{\left|{{}^{\circ}C}_{\kappa_{CB-1}}^{RN}\right|}^{∄}+℩\left(-\left({\left|{{}^{\circ}C}_{\kappa_{CB-1}}^{℩N}\right|}^{∄}\right)\right),\\ 1-{\left(1-\xi_{\kappa_{CB-1}}^{RP}\right)}^{∄}+℩\left(1-{\left(1-\xi_{\kappa_{CB-1}}^{℩P}\right)}^{∄}\right) \end{array}\right)$$

$${\;{∄\kappa }_{CB-1}}_{t}=\left(\begin{array}{c} 1-{\left(1-{{}^{\circ}C}_{\kappa_{CB-1}}^{RP}\right)}^{∄}+℩\left(1-{\left(1-{{}^{\circ}C}_{\kappa_{CB-1}}^{℩P}\right)}^{∄}\right),\\ -{\left|{{}^{\circ}C}_{\kappa_{CB-1}}^{RN}\right|}^{∄}+℩\left(-\left({\left|{{}^{\circ}C}_{\kappa_{CB-1}}^{℩N}\right|}^{∄}\right)\right)\\ {\left(\xi_{\kappa_{CB-1}}^{RP}\right)}^{∄}+℩{\left(\xi_{\kappa_{CB-1}}^{℩P}\right)}^{∄}, \end{array}\right)$$

$${{\kappa_{CB-1}}^{∄}}_{tc}=\left(\begin{array}{c} {\left({{}^{\circ}C}_{\kappa_{CB-1}}^{RP}\right)}^{∄}+℩{\left({{}^{\circ}C}_{\kappa_{CB-1}}^{℩P}\right)}^{∄},-1+{\left(1+{{}^{\circ}C}_{\kappa_{CB-1}}^{RN}\right)}^{∄}+℩\left(-1+{\left(1+{{}^{\circ}C}_{\kappa_{CB-1}}^{℩N}\right)}^{∄}\right)\\ {\left(\xi_{\kappa_{CB-1}}^{RP}\right)}^{∄}+℩{\left(\xi_{\kappa_{CB-1}}^{℩P}\right)}^{∄} \end{array},\right)$$

$${{\kappa_{CB-1}}^{∄}}_{t}=\left(\begin{array}{c} {\left({{}^{\circ}C}_{\kappa_{CB-1}}^{RP}\right)}^{∄}+℩{\left({{}^{\circ}C}_{\kappa_{CB-1}}^{℩P}\right)}^{∄},-1+{\left(1+{{}^{\circ}C}_{\kappa_{CB-1}}^{RN}\right)}^{∄}+℩\left(-1+{\left(1+{{}^{\circ}C}_{\kappa_{CB-1}}^{℩N}\right)}^{∄}\right)\\ 1-{\left(1-\xi_{\kappa_{CB-1}}^{RP}\right)}^{∄}+℩\left(1-{\left(1-\xi_{\kappa_{CB-1}}^{℩P}\right)}^{∄}\right) \end{array},\right)$$

## 3 Cir-BCFSs: circular bipolar complex fuzzy sets

In this section, we discussed Frank's operational laws and algebraic operational laws.

Definition 6 Assume $\kappa_{CB}=\left({{}^{\circ}C}_{\kappa_{CB}}^{P},{{}^{\circ}C}_{\kappa_{CB}}^{N},\xi_{\kappa_{CB}}^{P}\right)=\left({{}^{\circ}C}_{\kappa_{CB}}^{RP}+℩{{}^{\circ}C}_{\kappa_{CB}}^{℩P},{{}^{\circ}C}_{\kappa_{CB}}^{RN}+℩{{}^{\circ}C}_{\kappa_{CB}}^{℩N},\xi_{\kappa_{CB}}^{RP}+℩\xi_{\kappa_{CB}}^{℩P}\right)$ be a Cir-BCFN, then $S_{B}\left(\kappa_{CB}\right)=\frac{1}{6}\left({{}^{\circ}C}_{\kappa_{CB}}^{RP}+{{}^{\circ}C}_{\kappa_{CB}}^{℩P}+{{}^{\circ}C}_{\kappa_{CB}}^{RN}+{{}^{\circ}C}_{\kappa_{CB}}^{℩N}+\xi_{\kappa_{CB}}^{RP}+\xi_{\kappa_{CB}}^{℩P}\right),S_{B}\left(\kappa_{CB}\right)\in \left[O,1\right]$. Deduce the score value of $\kappa_{CB}$.

Definition 7 Assume $\kappa_{CB}=\left({{}^{\circ}C}_{\kappa_{CB}}^{P},{{}^{\circ}C}_{\kappa_{CB}}^{N},\xi_{\kappa_{CB}}^{P}\right)=\left({{}^{\circ}C}_{\kappa_{CB}}^{RP}+℩{{}^{\circ}C}_{\kappa_{CB}}^{℩P},{{}^{\circ}C}_{\kappa_{CB}}^{RN}+℩{{}^{\circ}C}_{\kappa_{CB}}^{℩N},\xi_{\kappa_{CB}}^{RP}+℩\xi_{\kappa_{CB}}^{℩P}\right)$ be a Cir-BCFN, then $H_{B}\left(\kappa_{CB}\right)=\frac{1}{6}\left({{}^{\circ}C}_{\kappa_{CB}}^{RP}+{{}^{\circ}C}_{\kappa_{CB}}^{℩P}-{{}^{\circ}C}_{\kappa_{CB}}^{RN}-{{}^{\circ}C}_{\kappa_{CB}}^{℩N}+\xi_{\kappa_{CB}}^{RP}+\xi_{\kappa_{CB}}^{℩P}\right),H_{B}\left(\kappa_{CB}\right)\in \left[O,1\right]$. Deduce the accuracy value of $\kappa_{CB}$.

Definition 8 Assume that $\kappa_{CB-1}=\left({{}^{\circ}C}_{\kappa_{CB-1}}^{P},{{}^{\circ}C}_{\kappa_{CB-1}}^{N},\xi_{\kappa_{CB-1}}^{P}\right)=\left({{}^{\circ}C}_{\kappa_{CB-1}}^{RP}+℩{{}^{\circ}C}_{\kappa_{CB-1}}^{℩P},{{}^{\circ}C}_{\kappa_{CB-1}}^{RN}+℩{{}^{\circ}C}_{\kappa_{CB-1}}^{℩N},\xi_{\kappa_{CB-1}}^{RP}+℩\xi_{\kappa_{CB-1}}^{℩P}\right)$ and $\kappa_{CB-2}=\left({{}^{\circ}C}_{\kappa_{CB-2}}^{P},{{}^{\circ}C}_{\kappa_{CB-2}}^{N},\xi_{\kappa_{CB-2}}^{P}\right)=\left({{}^{\circ}C}_{\kappa_{CB-2}}^{RP}+℩{{}^{\circ}C}_{\kappa_{CB-2}}^{℩P},{{}^{\circ}C}_{\kappa_{CB-2}}^{RN}+℩{{}^{\circ}C}_{\kappa_{CB-2}}^{℩N},\xi_{\kappa_{CB-2}}^{RP}+℩\xi_{\kappa_{CB-2}}^{℩P}\right)$ are two Cir-BCFNs ϜℲ>1, and ∄>O with and then Frank operational laws for Cir-BCFN, such as

$$\kappa_{CB-1}{⊕}_{tc}\;\kappa_{CB-2}=\left(\begin{array}{c} 1-\log_{ϜℲ}\left(1+\frac{\left({ϜℲ}^{1-{{}^{\circ}C}_{\kappa_{CB-1}}^{RP}}-1\right)\left({ϜℲ}^{1-{{}^{\circ}C}_{\kappa_{CB-2}}^{RP}}-1\right)}{ϜℲ-1}\right)\\ +℩\left(1-\log_{ϜℲ}\left(1+\frac{\left({ϜℲ}^{1-{{}^{\circ}C}_{\kappa_{CB-1}}^{℩P}}-1\right)\left({ϜℲ}^{1-{{}^{\circ}C}_{\kappa_{CB-2}}^{℩P}}-1\right)}{ϜℲ-1}\right)\right),\\ -\log_{ϜℲ}\left(1+\frac{\left({ϜℲ}^{-{{}^{\circ}C}_{\kappa_{CB-1}}^{RN}}-1\right)\left({ϜℲ}^{-{{}^{\circ}C}_{\kappa_{CB-2}}^{RN}}-1\right)}{ϜℲ-1}\right)\\ +℩\left(-\left(\log_{ϜℲ}\left(1+\frac{\left({ϜℲ}^{-{{}^{\circ}C}_{\kappa_{CB-1}}^{℩N}}-1\right)\left({ϜℲ}^{-{{}^{\circ}C}_{\kappa_{CB-2}}^{℩N}}-1\right)}{ϜℲ-1}\right)\right)\right),\\ 1-\log_{ϜℲ}\left(1+\frac{\left({ϜℲ}^{1-\xi_{\kappa_{CB-1}}^{RP}}-1\right)\left({ϜℲ}^{1-\xi_{\kappa_{CB-2}}^{RP}}-1\right)}{ϜℲ-1}\right)+\\ ℩\left(1-\log_{ϜℲ}\left(1+\frac{\left({ϜℲ}^{1-\xi_{\kappa_{CB-1}}^{℩P}}-1\right)\left({ϜℲ}^{1-\xi_{\kappa_{CB-2}}^{℩P}}-1\right)}{ϜℲ-1}\right)\right) \end{array}\right)$$

$$\kappa_{CB-1}{⊕}_{t}\;\kappa_{CB-2}=\left(\begin{array}{c} 1-\log_{ϜℲ}\left(1+\frac{\left({ϜℲ}^{1-{{}^{\circ}C}_{\kappa_{CB-1}}^{RP}}-1\right)\left({ϜℲ}^{1-{{}^{\circ}C}_{\kappa_{CB-2}}^{RP}}-1\right)}{ϜℲ-1}\right)\\ +℩\left(1-\log_{ϜℲ}\left(1+\frac{\left({ϜℲ}^{1-{{}^{\circ}C}_{\kappa_{CB-1}}^{℩P}}-1\right)\left({ϜℲ}^{1-{{}^{\circ}C}_{\kappa_{CB-2}}^{℩P}}-1\right)}{ϜℲ-1}\right)\right),\\ -\log_{ϜℲ}\left(1+\frac{\left({ϜℲ}^{-{{}^{\circ}C}_{\kappa_{CB-1}}^{RN}}-1\right)\left({ϜℲ}^{-{{}^{\circ}C}_{\kappa_{CB-2}}^{RN}}-1\right)}{ϜℲ-1}\right)\\ +℩\left(-\left(\log_{ϜℲ}\left(1+\frac{\left({ϜℲ}^{-{{}^{\circ}C}_{\kappa_{CB-1}}^{℩N}}-1\right)\left({ϜℲ}^{-{{}^{\circ}C}_{\kappa_{CB-2}}^{℩N}}-1\right)}{ϜℲ-1}\right)\right)\right),\\ \log_{ϜℲ}\left(1+\frac{\left({ϜℲ}^{\xi_{\kappa_{CB-1}}^{RP}}-1\right)\left({ϜℲ}^{\xi_{\kappa_{CB-2}}^{RP}}-1\right)}{ϜℲ-1}\right)+\\ ℩\left(\log_{ϜℲ}\left(1+\frac{\left({ϜℲ}^{\xi_{\kappa_{CB-1}}^{℩P}}-1\right)\left({ϜℲ}^{\xi_{\kappa_{CB-2}}^{℩P}}-1\right)}{ϜℲ-1}\right)\right) \end{array}\right)$$

$$\kappa_{CB-1}{⊗}_{tc}\kappa_{CB-2}=\left(\begin{array}{c} {\;\log}_{ϜℲ}\left(1+\frac{\left({ϜℲ}^{{{}^{\circ}C}_{\kappa_{CB-1}}^{RP}}-1\right)\left({ϜℲ}^{{{}^{\circ}C}_{\kappa_{CB-2}}^{RP}}-1\right)}{ϜℲ-1}\right)\\ +℩\left(\log_{ϜℲ}\left(1+\frac{\left({ϜℲ}^{{{}^{\circ}C}_{\kappa_{CB-1}}^{℩P}}-1\right)\left({ϜℲ}^{{{}^{\circ}C}_{\kappa_{CB-2}}^{℩P}}-1\right)}{ϜℲ-1}\right)\right),\\ -1+\log_{ϜℲ}\left(1+\frac{\left({ϜℲ}^{1+{{}^{\circ}C}_{\kappa_{CB-1}}^{RN}}-1\right)\left({ϜℲ}^{1+{{}^{\circ}C}_{\kappa_{CB-2}}^{RN}}-1\right)}{ϜℲ-1}\right)\\ +℩\left(-1+\log_{ϜℲ}\left(1+\frac{\left({ϜℲ}^{1+{{}^{\circ}C}_{\kappa_{CB-1}}^{℩N}}-1\right)\left({ϜℲ}^{1+{{}^{\circ}C}_{\kappa_{CB-2}}^{℩N}}-1\right)}{ϜℲ-1}\right)\right),\\ {\;\log}_{ϜℲ}\left(1+\frac{\left({ϜℲ}^{\xi_{\kappa_{CB-1}}^{RP}}-1\right)\left({ϜℲ}^{\xi_{\kappa_{CB-2}}^{RP}}-1\right)}{ϜℲ-1}\right)\\ +℩\left(\log_{ϜℲ}\left(1+\frac{\left({ϜℲ}^{\xi_{\kappa_{CB-1}}^{℩P}}-1\right)\left({ϜℲ}^{\xi_{\kappa_{CB-2}}^{℩P}}-1\right)}{ϜℲ-1}\right)\right) \end{array}\right)$$

$$\kappa_{CB-1}{⊗}_{t}\kappa_{CB-2}=\left(\begin{array}{c} {\;\log}_{ϜℲ}\left(1+\frac{\left({ϜℲ}^{{{}^{\circ}C}_{\kappa_{CB-1}}^{RP}}-1\right)\left({ϜℲ}^{{{}^{\circ}C}_{\kappa_{CB-2}}^{RP}}-1\right)}{ϜℲ-1}\right)\\ +℩\left(\log_{ϜℲ}\left(1+\frac{\left({ϜℲ}^{{{}^{\circ}C}_{\kappa_{CB-1}}^{℩P}}-1\right)\left({ϜℲ}^{{{}^{\circ}C}_{\kappa_{CB-2}}^{℩P}}-1\right)}{ϜℲ-1}\right)\right),\\ -1+\log_{ϜℲ}\left(1+\frac{\left({ϜℲ}^{1+{{}^{\circ}C}_{\kappa_{CB-1}}^{RN}}-1\right)\left({ϜℲ}^{1+{{}^{\circ}C}_{\kappa_{CB-2}}^{RN}}-1\right)}{ϜℲ-1}\right)\\ +℩\left(-1+\log_{ϜℲ}\left(1+\frac{\left({ϜℲ}^{1+{{}^{\circ}C}_{\kappa_{CB-1}}^{℩N}}-1\right)\left({ϜℲ}^{1+{{}^{\circ}C}_{\kappa_{CB-2}}^{℩N}}-1\right)}{ϜℲ-1}\right)\right),\\ 1-\log_{ϜℲ}\left(1+\frac{\left({ϜℲ}^{1-\xi_{\kappa_{CB-1}}^{RP}}-1\right)\left({ϜℲ}^{1-\xi_{\kappa_{CB-2}}^{RP}}-1\right)}{ϜℲ-1}\right)\\ +℩\left(1-\log_{ϜℲ}\left(1+\frac{\left({ϜℲ}^{1-\xi_{\kappa_{CB-1}}^{℩P}}-1\right)\left({ϜℲ}^{1-\xi_{\kappa_{CB-2}}^{℩P}}-1\right)}{ϜℲ-1}\right)\right) \end{array}\right)$$

$${{∄\kappa }_{CB-1}}_{tc}=\left(\begin{array}{c} 1-{\;\log}_{ϜℲ}\left(1+\frac{{\left({ϜℲ}^{1-{{}^{\circ}C}_{\kappa_{CB-1}}^{RP}}-1\right)}^{∄}}{{\left(ϜℲ-1\right)}^{∄-1}}\right)\\ +℩\left(1-\log_{ϜℲ}\left(1+\frac{{\left({ϜℲ}^{1-{{}^{\circ}C}_{\kappa_{CB-1}}^{℩P}}-1\right)}^{∄}}{{\left(ϜℲ-1\right)}^{∄-1}}\right)\right),\\ -\left({\;\log}_{ϜℲ}\left(1+\frac{{\left({ϜℲ}^{-{{}^{\circ}C}_{\kappa_{CB-1}}^{RN}}-1\right)}^{∄}}{{\left(ϜℲ-1\right)}^{∄-1}}\right)\right)\\ +℩\left(-\left({\;\log}_{ϜℲ}\left(1+\frac{{\left({ϜℲ}^{-{{}^{\circ}C}_{\kappa_{CB-1}}^{℩N}}-1\right)}^{∄}}{{\left(ϜℲ-1\right)}^{∄-1}}\right)\right)\right),\\ 1-{\;\log}_{ϜℲ}\left(1+\frac{{\left({ϜℲ}^{1-\xi_{\kappa_{CB-1}}^{RP}}-1\right)}^{∄}}{{\left(ϜℲ-1\right)}^{∄-1}}\right)\\ +℩\left(1-\log_{ϜℲ}\left(1+\frac{{\left({ϜℲ}^{1-\xi_{\kappa_{CB-1}}^{℩P}}-1\right)}^{∄}}{{\left(ϜℲ-1\right)}^{∄-1}}\right)\right) \end{array}\right)$$

$${{∄\kappa }_{CB-1}}_{t}=\left(\begin{array}{c} 1-{\;\log}_{ϜℲ}\left(1+\frac{{\left({ϜℲ}^{1-{{}^{\circ}C}_{\kappa_{CB-1}}^{RP}}-1\right)}^{∄}}{{\left(ϜℲ-1\right)}^{∄-1}}\right)\\ +℩\left(1-\log_{ϜℲ}\left(1+\frac{{\left({ϜℲ}^{1-{{}^{\circ}C}_{\kappa_{CB-1}}^{℩P}}-1\right)}^{∄}}{{\left(ϜℲ-1\right)}^{∄-1}}\right)\right),\\ -\left({\;\log}_{ϜℲ}\left(1+\frac{{\left({ϜℲ}^{-{{}^{\circ}C}_{\kappa_{CB-1}}^{RN}}-1\right)}^{∄}}{{\left(ϜℲ-1\right)}^{∄-1}}\right)\right)\\ +℩\left(-\left({\;\log}_{ϜℲ}\left(1+\frac{{\left({ϜℲ}^{-{{}^{\circ}C}_{\kappa_{CB-1}}^{℩N}}-1\right)}^{∄}}{{\left(ϜℲ-1\right)}^{∄-1}}\right)\right)\right),\\ {\;\log}_{ϜℲ}\left(1+\frac{{\left({ϜℲ}^{\xi_{\kappa_{CB-1}}^{RP}}-1\right)}^{∄}}{{\left(ϜℲ-1\right)}^{∄-1}}\right)\\ +℩\left(\log_{ϜℲ}\left(1+\frac{{\left({ϜℲ}^{\xi_{\kappa_{CB-1}}^{℩P}}-1\right)}^{∄}}{{\left(ϜℲ-1\right)}^{∄-1}}\right)\right) \end{array}\right)$$

$${{\kappa_{CB-1}}^{∄}}_{tc}=\left(\begin{array}{c} {\;\log}_{ϜℲ}\left(1+\frac{{\left({ϜℲ}^{{{}^{\circ}C}_{\kappa_{CB-1}}^{RP}}-1\right)}^{∄}}{{\left(ϜℲ-1\right)}^{∄-1}}\right)+℩\left(\log_{ϜℲ}\left(1+\frac{{\left({ϜℲ}^{{{}^{\circ}C}_{\kappa_{CB-1}}^{℩P}}-1\right)}^{∄}}{{\left(ϜℲ-1\right)}^{∄-1}}\right)\right),\\ -1+\left({\;\log}_{ϜℲ}\left(1+\frac{{\left({ϜℲ}^{1+{{}^{\circ}C}_{\kappa_{CB-1}}^{RN}}-1\right)}^{∄}}{{\left(ϜℲ-1\right)}^{∄-1}}\right)\right)+℩\left({-1+\log}_{ϜℲ}\left(1+\frac{{\left({ϜℲ}^{1+{{}^{\circ}C}_{\kappa_{CB-1}}^{℩N}}-1\right)}^{∄}}{{\left(ϜℲ-1\right)}^{∄-1}}\right)\right),\\ {\;\log}_{ϜℲ}\left(1+\frac{{\left({ϜℲ}^{\xi_{\kappa_{CB-1}}^{RP}}-1\right)}^{∄}}{{\left(ϜℲ-1\right)}^{∄-1}}\right)+℩\left(\log_{ϜℲ}\left(1+\frac{{\left({ϜℲ}^{\xi_{\kappa_{CB-1}}^{℩P}}-1\right)}^{∄}}{{\left(ϜℲ-1\right)}^{∄-1}}\right)\right) \end{array}\right)$$

$${{\kappa_{CB-1}}^{∄}}_{t}=\left(\begin{array}{c} {\;\log}_{ϜℲ}\left(1+\frac{{\left({ϜℲ}^{{{}^{\circ}C}_{\kappa_{CB-1}}^{RP}}-1\right)}^{∄}}{{\left(ϜℲ-1\right)}^{∄-1}}\right)+℩\left(\log_{ϜℲ}\left(1+\frac{{\left({ϜℲ}^{{{}^{\circ}C}_{\kappa_{CB-1}}^{℩P}}-1\right)}^{∄}}{{\left(ϜℲ-1\right)}^{∄-1}}\right)\right),\\ -1+\left({\;\log}_{ϜℲ}\left(1+\frac{{\left({ϜℲ}^{1+{{}^{\circ}C}_{\kappa_{CB-1}}^{RN}}-1\right)}^{∄}}{{\left(ϜℲ-1\right)}^{∄-1}}\right)\right)+℩\left({-1+\log}_{ϜℲ}\left(1+\frac{{\left({ϜℲ}^{1+{{}^{\circ}C}_{\kappa_{CB-1}}^{℩N}}-1\right)}^{∄}}{{\left(ϜℲ-1\right)}^{∄-1}}\right)\right),\\ 1-{\;\log}_{ϜℲ}\left(1+\frac{{\left({ϜℲ}^{1-\xi_{\kappa_{CB-1}}^{RP}}-1\right)}^{∄}}{{\left(ϜℲ-1\right)}^{∄-1}}\right)+℩\left(1-\log_{ϜℲ}\left(1+\frac{{\left({ϜℲ}^{1-\xi_{\kappa_{CB-1}}^{℩P}}-1\right)}^{∄}}{{\left(ϜℲ-1\right)}^{∄-1}}\right)\right) \end{array}\right)$$

## 4 Frank power aggregation operators for Cir-BCFSs

This section introduced the technique of the Cir-BCFFPA operator, Cir-BCFFPWA operator, Cir-BCFFPG operator, and Cir-BCFFPWG operator with some properties, called idempotency, monotonicity, and boundedness.

Definition 9 Let's consider Cir-BCFNs i. e $\kappa_{CB-\varepsilon }=\left({{}^{\circ}C}_{\kappa_{CB-\varepsilon }}^{P},{{}^{\circ}C}_{\kappa_{CB-\varepsilon }}^{N},\xi_{\kappa_{CB-\varepsilon }}^{P}\right)=\left({{}^{\circ}C}_{\kappa_{CB-\varepsilon }}^{RP}+℩{{}^{\circ}C}_{\kappa_{CB-\varepsilon }}^{℩P},{{}^{\circ}C}_{\kappa_{CB-\varepsilon }}^{RN}+℩{{}^{\circ}C}_{\kappa_{CB-\varepsilon }}^{℩N},\xi_{\kappa_{CB}-\varepsilon }^{RP}+℩\xi_{\kappa_{CB-\varepsilon }}^{℩P}\right)$, $\varepsilon =1,2,3,\ldots ,\ddot{u}$, then

$$Cir-BCFFPA{\left(\kappa_{CB-1},\kappa_{CB-2},\ldots ,\kappa_{CB-u\;}\right)}_{tc}={{⊕}_{\varepsilon =1}^{u}}_{tc}\frac{\left(1+T\left(\kappa_{CB-\varepsilon }\right)\right)}{\sum_{\varepsilon =1}^{.u}\left(1+T\left(\kappa_{CB-\varepsilon }\right)\right)}\kappa_{CB-\varepsilon }$$

$$Cir-BCFFPA{\left(\kappa_{CB-1},\kappa_{CB-2},\ldots ,\kappa_{CB-\ddot{u}\;}\right)}_{t}={{⊕}_{\varepsilon =1}^{u}}_{t}\frac{\left(1+T\left(\kappa_{CB-\varepsilon }\right)\right)}{\sum_{\varepsilon =1}^{.u}\left(1+T\left(\kappa_{CB-\varepsilon }\right)\right)}\kappa_{CB-\varepsilon }$$

is defined as Cir-BCFFFPA operator for both t-norm and t-conorm with $T\left(\kappa_{CB-\varepsilon }\right)=\sum_{\begin{array}{c} i=1\\ i\neq \varepsilon \end{array}}^{u}\;\mathrm{Sup}\left(\kappa_{CB-\varepsilon },\kappa_{CB-i}\right)$ and $\mathrm{Sup}\left(\kappa_{CB-\varepsilon },\kappa_{CB-i}\right)=1-Dis\left(\kappa_{CB-\varepsilon },\kappa_{CB-i}\right)$.

Theorem 1 *Consider a set of Cir-BCFNs*, *the aggregated value would be a Cir-BCFN*, *such as*

$${Cir-BCFFPA}_{tc}\left(\kappa_{CB-1},\kappa_{CB-2},\ldots ,\kappa_{CB-u\;}\right)=\left(\begin{array}{c} 1-\log_{ϜℲ}\left(1+{\prod_{\varepsilon =1}^{u}\left({ϜℲ}^{1-{{}^{\circ}C}_{\kappa_{CB-\varepsilon }}^{RP}}-1\right)}^{\frac{\left(1+T\left(\kappa_{CB-\varepsilon }\right)\right)}{\sum_{\varepsilon =1}^{.\ddot{u}}\left(1+T\left(\kappa_{CB-\varepsilon }\right)\right)}}\right)\\ +℩\left(1-\log_{ϜℲ}\left(1+\prod_{\varepsilon =1}^{u}{\left({ϜℲ}^{1-{{}^{\circ}C}_{\kappa_{CB-\varepsilon }}^{℩P}}-1\right)}^{\frac{\left(1+T\left(\kappa_{CB-\varepsilon }\right)\right)}{\sum_{\varepsilon =1}^{.\ddot{u}}\left(1+T\left(\kappa_{CB-\varepsilon }\right)\right)}}\right)\right),\\ -\left(\log_{ϜℲ}\left(1+\prod_{\varepsilon =1}^{u}{\left({ϜℲ}^{-{{}^{\circ}C}_{\kappa_{CB-\varepsilon }}^{RN}}-1\right)}^{\frac{\left(1+T\left(\kappa_{CB-\varepsilon }\right)\right)}{\sum_{\varepsilon =1}^{.\ddot{u}}\left(1+T\left(\kappa_{CB-\varepsilon }\right)\right)}}\right)\right)\\ +℩\left(-\left(\log_{ϜℲ}\left(1+\prod_{\varepsilon =1}^{u}{\left({ϜℲ}^{-{{}^{\circ}C}_{\kappa_{CB-\varepsilon }}^{℩N}}-1\right)}^{\frac{\left(1+T\left(\kappa_{CB-\varepsilon }\right)\right)}{\sum_{\varepsilon =1}^{.\ddot{u}}\left(1+T\left(\kappa_{CB-\varepsilon }\right)\right)}}\right)\right)\right),\\ 1-\log_{ϜℲ}\left(1+\prod_{\varepsilon =1}^{u}{\left({ϜℲ}^{1-\xi_{\kappa_{CB-\varepsilon }}^{RP}}-1\right)}^{\frac{\left(1+T\left(\kappa_{CB-\varepsilon }\right)\right)}{\sum_{\varepsilon =1}^{.\ddot{u}}\left(1+T\left(\kappa_{CB-\varepsilon }\right)\right)}}\right)\\ +℩\left(1-\log_{ϜℲ}\left(1+\prod_{\varepsilon =1}^{u}{\left({ϜℲ}^{1-\xi_{\kappa_{CB-\varepsilon }}^{℩P}}-1\right)}^{\frac{\left(1+T\left(\kappa_{CB-\varepsilon }\right)\right)}{\sum_{\varepsilon =1}^{.\ddot{u}}\left(1+T\left(\kappa_{CB-\varepsilon }\right)\right)}}\right)\right) \end{array}\right)$$

$${Cir-BCFFPA}_{t}\left(\kappa_{CB-1},\kappa_{CB-2},\ldots ,\kappa_{CB-u\;}\right)=\left(\begin{array}{c} 1-\log_{ϜℲ}\left(1+\prod_{\varepsilon =1}^{u}{\left({ϜℲ}^{1-{{}^{\circ}C}_{\kappa_{CB-\varepsilon }}^{RP}}-1\right)}^{\frac{\left(1+T\left(\kappa_{CB-\varepsilon }\right)\right)}{\sum_{\varepsilon =1}^{.\ddot{u}}\left(1+T\left(\kappa_{CB-\varepsilon }\right)\right)}}\right)\\ +℩\left(1-\log_{ϜℲ}\left(1+\prod_{\varepsilon =1}^{u}{\left({ϜℲ}^{1-{{}^{\circ}C}_{\kappa_{CB-\varepsilon }}^{℩P}}-1\right)}^{\frac{\left(1+T\left(\kappa_{CB-\varepsilon }\right)\right)}{\sum_{\varepsilon =1}^{.\ddot{u}}\left(1+T\left(\kappa_{CB-\varepsilon }\right)\right)}}\right)\right),\\ -\left(\log_{ϜℲ}\left(1+\prod_{\varepsilon =1}^{u}{\left({ϜℲ}^{-{{}^{\circ}C}_{\kappa_{CB-\varepsilon }}^{RN}}-1\right)}^{\frac{\left(1+T\left(\kappa_{CB-\varepsilon }\right)\right)}{\sum_{\varepsilon =1}^{.\ddot{u}}\left(1+T\left(\kappa_{CB-\varepsilon }\right)\right)}}\right)\right)\\ +℩\left(-\left(\log_{ϜℲ}\left(1+{\prod_{\varepsilon =1}^{u}\left({ϜℲ}^{-{{}^{\circ}C}_{\kappa_{CB-\varepsilon }}^{℩N}}-1\right)}^{\frac{\left(1+T\left(\kappa_{CB-\varepsilon }\right)\right)}{\sum_{\varepsilon =1}^{.\ddot{u}}\left(1+T\left(\kappa_{CB-\varepsilon }\right)\right)}}\right)\right)\right),\\ \left(\log_{ϜℲ}\left(1+\prod_{\varepsilon =1}^{u}{\left({ϜℲ}^{\xi_{\kappa_{CB-\varepsilon }}^{RP}}-1\right)}^{\frac{\left(1+T\left(\kappa_{CB-\varepsilon }\right)\right)}{\sum_{\varepsilon =1}^{.\ddot{u}}\left(1+T\left(\kappa_{CB-\varepsilon }\right)\right)}}\right)\right)\\ +℩\left(\log_{ϜℲ}\left(1+\prod_{\varepsilon =1}^{u}{\left({ϜℲ}^{\xi_{\kappa_{CB-\varepsilon }}^{℩P}}-1\right)}^{\frac{\left(1+T\left(\kappa_{CB-\varepsilon }\right)\right)}{\sum_{\varepsilon =1}^{.\ddot{u}}\left(1+T\left(\kappa_{CB-\varepsilon }\right)\right)}}\right)\right) \end{array}\right)$$

Proof *Using mathematical induction*, *we prove that the above operators*. *Let*

$${Cir-BCFFPA}_{tc}\left(\kappa_{CB-1}\right)⊕{Cir-BCFFPA}_{tc}\left(\kappa_{CB-2}\right)=\left(\begin{array}{c} \begin{array}{c} 1-\log_{ϜℲ}\left(1+\frac{{\left({ϜℲ}^{1-{{}^{\circ}C}_{\kappa_{CB-\varepsilon }}^{RP}}-1\right)}^{A_{1}}}{{\left(ϜℲ-1\right)}^{A_{D+1}-1}}\right)+℩\left(1-\log_{ϜℲ}\left(1+\frac{{\left({ϜℲ}^{1-{{}^{\circ}C}_{BCBFS-1}^{℩P}}-1\right)}^{A_{1}}}{{\left(ϜℲ-1\right)}^{A_{D+1}-1}}\right)\right),-\log_{ϜℲ}\left(1+\frac{{\left({ϜℲ}^{-{{}^{\circ}C}_{\kappa_{CB-\varepsilon }}^{℩N}}-1\right)}^{A_{1}}}{{\left(ϜℲ-1\right)}^{A_{1}-1}}\right)+℩\left(-\left(\log_{ϜℲ}\left(\begin{array}{c} 1+\\ \frac{{\left({ϜℲ}^{-{{}^{\circ}C}_{\kappa_{CB-\varepsilon }}^{℩N}}-1\right)}^{A_{1}}}{{\left(ϜℲ-1\right)}^{A_{1}-1}} \end{array}\right)\right)\right),\\ 1-\log_{ϜℲ}\left(1+\frac{{\left({ϜℲ}^{1-\xi_{\kappa_{CB-\varepsilon }}^{RP}}-1\right)}^{A_{1}}}{{\left(ϜℲ-1\right)}^{A_{1}-1}}\right) \end{array}\\ +℩\left(1-\log_{ϜℲ}\left(1+\frac{{\left({ϜℲ}^{1-\xi_{\kappa_{CB-\varepsilon }}^{℩P}}-1\right)}^{A_{1}}}{{\left(ϜℲ-1\right)}^{A_{1}-1}}\right)\right) \end{array}\right)⊕\left(\begin{array}{c} \begin{array}{c} 1-\log_{ϜℲ}\left(1+\frac{{\left({ϜℲ}^{1-{{}^{\circ}C}_{\kappa_{CB-\varepsilon }}^{RP}}-1\right)}^{A_{2}}}{{\left(ϜℲ-1\right)}^{A_{2}-1}}\right)+℩\left(1-\log_{ϜℲ}\left(1+\frac{{\left({ϜℲ}^{1-{{}^{\circ}C}_{\kappa_{CB-\varepsilon }}^{℩P}}-1\right)}^{A_{2}}}{{\left(ϜℲ-1\right)}^{A_{2}-1}}\right)\right),-\log_{ϜℲ}\left(1+\frac{{\left({ϜℲ}^{-{{}^{\circ}C}_{\kappa_{CB-\varepsilon }}^{℩N}}-1\right)}^{A_{2}}}{{\left(ϜℲ-1\right)}^{A_{2}-1}}\right)+℩\left(-\left(\log_{ϜℲ}\left(1+\frac{{\left({ϜℲ}^{-{{}^{\circ}C}_{\kappa_{CB-\varepsilon }}^{℩N}}-1\right)}^{A_{2}}}{{\left(ϜℲ-1\right)}^{A_{2}-1}}\right)\right)\right),\\ 1-\log_{ϜℲ}\left(1+\frac{{\left({ϜℲ}^{1-\xi_{\kappa_{CB-\varepsilon }}^{RP}}-1\right)}^{A_{2}}}{{\left(ϜℲ-1\right)}^{A_{2}-1}}\right) \end{array}\\ +℩\left(1-\log_{ϜℲ}\left(1+\frac{{\left({ϜℲ}^{1-\xi_{\kappa_{CB-\varepsilon }}^{℩P}}-1\right)}^{A_{2}}}{{\left(ϜℲ-1\right)}^{A_{2}-1}}\right)\right) \end{array}\right)$$

$${Cir-BCFFPA}_{tc}\left(\kappa_{CB-1}\right)⊕{Cir-BCFFPA}_{tc}\left(\kappa_{CB-2}\right)=\left(\begin{array}{c} 1-\log_{ϜℲ}\left(1+\prod_{\varepsilon =1}^{2}{\left({ϜℲ}^{1-{{}^{\circ}C}_{\kappa_{CB-\varepsilon }}^{RP}}-1\right)}^{A_{\varepsilon }}\right)\\ +℩\left(1-\log_{ϜℲ}\left(1+\prod_{\varepsilon =1}^{2}{\left({ϜℲ}^{1-{{}^{\circ}C}_{\kappa_{CB-\varepsilon }}^{℩P}}-1\right)}^{A_{\varepsilon }}\right)\right),\\ -\left(\log_{ϜℲ}\left(1+\prod_{\varepsilon =1}^{2}{\left({ϜℲ}^{-{{}^{\circ}C}_{\kappa_{CB-\varepsilon }}^{RN}}-1\right)}^{A_{\varepsilon }}\right)\right)\\ +℩\left(-\left(\log_{ϜℲ}\left(1+\prod_{\varepsilon =1}^{2}{\left({ϜℲ}^{-{{}^{\circ}C}_{\kappa_{CB-\varepsilon }}^{℩N}}-1\right)}^{A_{\varepsilon }}\right)\right)\right),\\ 1-\log_{ϜℲ}\left(1+\prod_{\varepsilon =1}^{2}{\left({ϜℲ}^{1-\xi_{\kappa_{CB-\varepsilon }}^{RP}}-1\right)}^{A_{\varepsilon }}\right)\\ +℩\left(1-\log_{ϜℲ}\left(1+{\prod_{\varepsilon =1}^{2}\left({ϜℲ}^{1-\xi_{\kappa_{CB-\varepsilon }}^{℩P}}-1\right)}^{A_{\varepsilon }}\right)\right) \end{array}\right)$$

*Suppose the given equation holds for*u=D.

$${Cir-BCFFPA}_{tc}\left(\kappa_{CB-1},\kappa_{CB-2},\ldots ,\kappa_{CB-D}\right)=\left(\begin{array}{c} 1-\log_{ϜℲ}\left(1+\prod_{\varepsilon =1}^{D}{\left({ϜℲ}^{1-{{}^{\circ}C}_{\kappa_{CB-\varepsilon }}^{RP}}-1\right)}^{A_{\varepsilon }}\right)\\ +℩\left(1-\log_{ϜℲ}\left(1+\prod_{\varepsilon =1}^{D}{\left({ϜℲ}^{1-{{}^{\circ}C}_{\kappa_{CB-\varepsilon }}^{℩P}}-1\right)}^{A_{\varepsilon }}\right)\right),\\ -\left(\log_{ϜℲ}\left(1+{\prod_{\varepsilon =1}^{D}\left({ϜℲ}^{-{{}^{\circ}C}_{\kappa_{CB-\varepsilon }}^{RN}}-1\right)}^{A_{\varepsilon }}\right)\right)\\ +℩\left(-\left(\log_{ϜℲ}\left(1+{\prod_{\varepsilon =1}^{D}\left({ϜℲ}^{-{{}^{\circ}C}_{\kappa_{CB-\varepsilon }}^{℩N}}-1\right)}^{A_{\varepsilon }}\right)\right)\right),\\ 1-\log_{ϜℲ}\left(1+\prod_{\varepsilon =1}^{D}{\left({ϜℲ}^{1-\xi_{\kappa_{CB-\varepsilon }}^{RP}}-1\right)}^{A_{\varepsilon }}\right)\\ +℩\left(1-\log_{ϜℲ}\left(1+\prod_{\varepsilon =1}^{D}{\left({ϜℲ}^{1-\xi_{\kappa_{CB-\varepsilon }}^{℩P}}-1\right)}^{A_{\varepsilon }}\right)\right) \end{array}\right)$$

*Now we will prove that the given equation is true for*u=D+1, *such as*

$${Cir-BCFFPA}_{tc}\left(\kappa_{CB-1},\kappa_{CB-2},\ldots ,\kappa_{CB-\left(D+1\right)}\right)=\left(\begin{array}{c} 1-\log_{ϜℲ}\left(1+\prod_{\varepsilon =1}^{D}{\left({ϜℲ}^{1-{{}^{\circ}C}_{\kappa_{CB-\varepsilon }}^{RP}}-1\right)}^{A_{\varepsilon }}\right)\\ +℩\left(1-\log_{ϜℲ}\left(1+\prod_{\varepsilon =1}^{D}{\left({ϜℲ}^{1-{{}^{\circ}C}_{\kappa_{CB-\varepsilon }}^{℩P}}-1\right)}^{A_{\varepsilon }}\right)\right),\\ -\left(\log_{ϜℲ}\left(1+\prod_{\varepsilon =1}^{D}{\left({ϜℲ}^{-{{}^{\circ}C}_{\kappa_{CB-\varepsilon }}^{RN}}-1\right)}^{A_{\varepsilon }}\right)\right)\\ +℩\left(-\left(\log_{ϜℲ}\left(1+\prod_{\varepsilon =1}^{D}{\left({ϜℲ}^{-{{}^{\circ}C}_{\kappa_{CB-\varepsilon }}^{℩N}}-1\right)}^{A_{\varepsilon }}\right)\right)\right),\\ 1-\log_{ϜℲ}\left(1+\prod_{\varepsilon =1}^{D}{\left({ϜℲ}^{1-\xi_{\kappa_{CB-\varepsilon }}^{RP}}-1\right)}^{A_{\varepsilon }}\right)\\ +℩\left(1-\log_{ϜℲ}\left(1+\prod_{\varepsilon =1}^{D}{\left({ϜℲ}^{1-\xi_{\kappa_{CB-\varepsilon }}^{℩P}}-1\right)}^{A_{\varepsilon }}\right)\right) \end{array}\right)⊕\left(\begin{array}{c} \begin{array}{c} 1-\log_{ϜℲ}\left(1+\frac{{\left({ϜℲ}^{1-{{}^{\circ}C}_{\kappa_{CB-\left(D+1\right)}}^{RP}}-1\right)}^{A_{D+1}}}{{\left(ϜℲ-1\right)}^{A_{D+1}-1}}\right)+℩\left(1-\log_{ϜℲ}\left(1+\frac{{\left({ϜℲ}^{1-{{}^{\circ}C}_{\kappa_{CB-\left(D+1\right)}}^{℩P}}-1\right)}^{A_{D+1}}}{{\left(ϜℲ-1\right)}^{A_{D+1}-1}}\right)\right),-\log_{ϜℲ}\left(1+\frac{{\left({ϜℲ}^{-{{}^{\circ}C}_{\kappa_{CB-\left(D+1\right)}}^{RN}}-1\right)}^{A_{D+1}}}{{\left(ϜℲ-1\right)}^{A_{D+1}-1}}\right)+℩\left(-\left(\log_{ϜℲ}\left(1+\frac{{\left({ϜℲ}^{-{{}^{\circ}C}_{\kappa_{CB-\left(D+1\right)}}^{℩N}}-1\right)}^{A_{D+1}}}{{\left(ϜℲ-1\right)}^{A_{D+1}-1}}\right)\right)\right),\\ 1-\log_{ϜℲ}\left(1+\frac{{\left({ϜℲ}^{1-\xi_{\kappa_{CB-\left(D+1\right)}}^{RP}}-1\right)}^{A_{D+1}}}{{\left(ϜℲ-1\right)}^{A_{D+1}-1}}\right) \end{array}\\ +℩\left(1-\log_{ϜℲ}\left(1+\frac{{\left({ϜℲ}^{1-\xi_{\kappa_{CB-\left(D+1\right)}}^{℩P}}-1\right)}^{A_{D+1}}}{{\left(ϜℲ-1\right)}^{A_{D+1}-1}}\right)\right) \end{array}\right)=\left(\begin{array}{c} 1-\log_{ϜℲ}\left(1+\prod_{\varepsilon =1}^{D+1}{\left({ϜℲ}^{1-{{}^{\circ}C}_{\kappa_{CB-\varepsilon }}^{RP}}-1\right)}^{A_{\varepsilon }}\right)\\ +℩\left(1-\log_{ϜℲ}\left(1+\prod_{\varepsilon =1}^{D+1}{\left({ϜℲ}^{1-{{}^{\circ}C}_{\kappa_{CB-\varepsilon }}^{℩P}}-1\right)}^{A_{\varepsilon }}\right)\right),\\ -\left(\log_{ϜℲ}\left(1+\prod_{\varepsilon =1}^{D+1}{\left({ϜℲ}^{-{{}^{\circ}C}_{\kappa_{CB-\varepsilon }}^{RN}}-1\right)}^{A_{\varepsilon }}\right)\right)\\ +℩\left(-\left(\log_{ϜℲ}\left(1+\prod_{\varepsilon =1}^{D+1}{\left({ϜℲ}^{-{{}^{\circ}C}_{\kappa_{CB-\varepsilon }}^{℩N}}-1\right)}^{A_{\varepsilon }}\right)\right)\right),\\ 1-\log_{ϜℲ}\left(1+{\prod_{\varepsilon =1}^{D+1}\left({ϜℲ}^{1-\xi_{\kappa_{CB-\varepsilon }}^{RP}}-1\right)}^{A_{\varepsilon }}\right)\\ +℩\left(1-\log_{ϜℲ}\left(1+{\prod_{\varepsilon =1}^{D+1}\left({ϜℲ}^{1-\xi_{\kappa_{CB-\varepsilon }}^{℩P}}-1\right)}^{A_{\varepsilon }}\right)\right) \end{array}\right)$$

*So*, *this implies that this equation holds for*u=D+1,*so the result is true for all u*.

*Assume two sets of Cir-BCFNs i*.*e*. $\kappa_{CB-1}=\left({{}^{\circ}C}_{\kappa_{CB-1}}^{P},{{}^{\circ}C}_{\kappa_{CB-1}}^{N},\xi_{\kappa_{CB-1}}^{P}\right)=\left({{}^{\circ}C}_{\kappa_{CB-1}}^{RP}+℩{{}^{\circ}C}_{\kappa_{CB-1}}^{℩P},{{}^{\circ}C}_{\kappa_{CB-1}}^{RN}+℩{{}^{\circ}C}_{\kappa_{CB-1}}^{℩N},\xi_{\kappa_{CB-1}}^{RP}+℩\xi_{\kappa_{CB-1}}^{℩P}\right)$*and*$\kappa_{CB-2}=\left({{}^{\circ}C}_{\kappa_{CB-2}}^{P},{{}^{\circ}C}_{\kappa_{CB-2}}^{N},\xi_{\kappa_{CB-2}}^{P}\right)=\left({{}^{\circ}C}_{\kappa_{CB-2}}^{RP}+℩{{}^{\circ}C}_{\kappa_{CB-2}}^{℩P},{{}^{\circ}C}_{\kappa_{CB-2}}^{RN}+℩{{}^{\circ}C}_{\kappa_{CB-2}}^{℩N},\xi_{\kappa_{CB-2}}^{RP}+℩\xi_{\kappa_{CB-2}}^{℩P}\right)$*then Cir-BFFPA operator has underneath properties*.

Property 1 *Idempotency*: ∀ε*if*$\kappa_{CB-\varepsilon }=\kappa_{CB}$, *then*

$${Cir-BCFFPA}_{tc}\left(\kappa_{CB-1},\kappa_{CB-2},\ldots ,\kappa_{CB-\ddot{u}}\right)=\kappa_{CB}$$

$${Cir-BCFFPA}_{t}\left(\kappa_{CB-1},\kappa_{CB-2},\ldots ,\kappa_{CB-\ddot{u}}\right)=\kappa_{CB}$$

Property 2 *Boundedness*: *Assume that for t-conorm we have*, $\kappa_{CB}=\left(\min_{\varepsilon }\left({{}^{\circ}C}_{\kappa_{CB-\varepsilon }}^{RP}\right)+℩\min_{\varepsilon }\left({{}^{\circ}C}_{\kappa_{CB-\varepsilon }}^{℩P}\right),\max_{\varepsilon }\left({{}^{\circ}C}_{\kappa_{CB-\varepsilon }}^{RN}\right)+℩\max_{\varepsilon }\left({{}^{\circ}C}_{\kappa_{CB-\varepsilon }}^{℩N}\right),\min_{\varepsilon }\left(\xi_{\kappa_{CB-\varepsilon }}^{RP}\right)+℩\min_{\varepsilon }\left(\xi_{\kappa_{CB-\varepsilon }}^{℩P}\right)\right)$*and*$\kappa_{CB}^{+}=\left(\max_{\varepsilon }\left({{}^{\circ}C}_{\kappa_{CB-\varepsilon }}^{RP}\right)+℩\max_{\varepsilon }\left({{}^{\circ}C}_{\kappa_{CB-\varepsilon }}^{℩P}\right),\min_{\varepsilon }\left({{}^{\circ}C}_{\kappa_{CB-\varepsilon }}^{RN}\right)+℩\min_{\varepsilon }\left({{}^{\circ}C}_{\kappa_{CB-\varepsilon }}^{℩N}\right),\max_{\varepsilon }\left(\xi_{\kappa_{CB-\varepsilon }}^{RP}\right)+℩\max_{\varepsilon }\left(\xi_{\kappa_{CB-\varepsilon }}^{℩P}\right)\right)$*and similarly for t-norm*, *so then*

$$\kappa_{CB}^{-}\leq {Cir-BCFFPA}_{tc}\left(\kappa_{CB-1},\kappa_{CB-2},\ldots ,\kappa_{CB-\ddot{u}}\right)\leq \kappa_{CB}^{+}$$

$$\kappa_{CB}^{-}\leq {Cir-BCFFPA}_{t}\left(\kappa_{CB-1},\kappa_{CB-2},\ldots ,\kappa_{CB-\ddot{u}}\right)\leq \kappa_{CB}^{+}$$

Property 3 *Monotonicity*: ∀ε*if*$\kappa_{CB-\varepsilon }\leq K_{CB}^{/}$, *then*

$${Cir-BCFFPA}_{tc}\left(\kappa_{CB-1},\kappa_{CB-2},\ldots ,\kappa_{CB-u}\right)\leq {Cir-BCFFPA}_{tc}\left(K_{CB-1}^{/},K_{CB-2}^{/},\ldots ,K_{CB-u}^{/}\right)$$

$${Cir-BCFFPA}_{t}\left(\kappa_{CB-1},\kappa_{CB-2},\ldots ,\kappa_{CB-u}\right)\leq {Cir-BCFFPA}_{t}\left(K_{CB-1}^{/},K_{CB-2}^{/},\ldots ,K_{CB-u}^{/}\right)$$

*There are various things in life in which we have to represent weight vectors provided by decision-makers*. *So*, *we introduce the new operator Cir-BCFFPWA operator*.

Definition 10 *Let's consider Cir-BCFNs i*. *e*$\kappa_{CB-\varepsilon }=\left({{}^{\circ}C}_{\kappa_{CB-\varepsilon }}^{P},{{}^{\circ}C}_{\kappa_{CB-\varepsilon }}^{N},\xi_{\kappa_{CB-\varepsilon }}^{P}\right)=\left({{}^{\circ}C}_{\kappa_{CB-\varepsilon }}^{RP}+℩{{}^{\circ}C}_{\kappa_{CB-\varepsilon }}^{℩P},{{}^{\circ}C}_{\kappa_{CB-\varepsilon }}^{RN}+℩{{}^{\circ}C}_{\kappa_{CB-\varepsilon }}^{℩N},\xi_{\kappa_{CB}-\varepsilon }^{RP}+℩\xi_{\kappa_{CB-\varepsilon }}^{℩P}\right)$, $\varepsilon =1,2,3,\ldots ,\ddot{u}$, *then*

$${Cir-BCFFPWA}_{tc}\left(\kappa_{CB-1},\kappa_{CB-2},\ldots ,\kappa_{CB-u\;}\right)={{⊕}_{\varepsilon =1}^{u}}_{tc}\frac{W_{vy-1}\left(1+T\left(\kappa_{CB-\varepsilon }\right)\right)}{\sum_{\varepsilon =1}^{.u}W_{vy-1}\left(1+T\left(\kappa_{CB-\varepsilon }\right)\right)}\kappa_{CB-\varepsilon }$$

$${Cir-BCFFPWA}_{t}\left(\kappa_{CB-1},\kappa_{CB-2},\ldots ,\kappa_{CB-u\;}\right)={{⊕}_{\varepsilon =1}^{u}}_{t}\frac{W_{vy-1}\left(1+T\left(\kappa_{CB-\varepsilon }\right)\right)}{\sum_{\varepsilon =1}^{.u}W_{vy-1}\left(1+T\left(\kappa_{CB-\varepsilon }\right)\right)}\kappa_{CB-\varepsilon }$$

*is defined as Cir-BCFFPWA operator for t-norm and t-conorm with*$T\left(\kappa_{CB-\varepsilon }\right)=\sum_{\begin{array}{c} i=1\\ i\neq \varepsilon \end{array}}^{u}\;\mathrm{Sup}\left(\kappa_{CB-\varepsilon },\kappa_{CB-i}\right),W_{vy-1}=\left(W_{vy-1},W_{vy-2},\ldots ,W_{vy-u}\right)$*is a weight vector* (*WV*) *satisfying that* $\sum_{\varepsilon =1}^{u}W_{vy-\emptyset }$, *and* $O\leq W_{vy-1}\leq 1\;\forall \varepsilon$.

Theorem 2 *Assume two sets of Cir-BCFNs i*. *e*. $\kappa_{CB-\varepsilon }=\left({{}^{\circ}C}_{\kappa_{CB-\varepsilon }}^{P},{{}^{\circ}C}_{\kappa_{CB-\varepsilon }}^{N},\xi_{\kappa_{CB-\varepsilon }}^{P}\right)=\left({{}^{\circ}C}_{\kappa_{CB-\varepsilon }}^{RP}+℩{{}^{\circ}C}_{\kappa_{CB-\varepsilon }}^{℩P},{{}^{\circ}C}_{\kappa_{CB-\varepsilon }}^{RN}+℩{{}^{\circ}C}_{\kappa_{CB-\varepsilon }}^{℩N},\xi_{\kappa_{CB-\varepsilon }}^{RP}+℩\xi_{\kappa_{CB-\varepsilon }}^{℩P}\right)$*and*$\kappa_{CB-\varepsilon }^{/}=\left({{}^{\circ}C}_{\kappa_{CB-\varepsilon }}^{P},{{}^{\circ}C}_{\kappa_{CB-\varepsilon }}^{N},\xi_{\kappa_{CB-\varepsilon }}^{P}\right)=\left({{}^{\circ}C}_{\kappa_{CB-\varepsilon }}^{RP}+℩{{}^{\circ}C}_{\kappa_{CB-\varepsilon }}^{℩P},{{}^{\circ}C}_{\kappa_{CB-\varepsilon }}^{RN}+℩{{}^{\circ}C}_{\kappa_{CB-\varepsilon }}^{℩N},\xi_{\kappa_{CB-\varepsilon }}^{RP}+℩\xi_{\kappa_{CB-\varepsilon }}^{℩P}\right)$, *then by employing the above Equation*, *the aggregated value would be a Cir-BCFN and*

$${Cir-BCFFPWA}_{tc}\left(\kappa_{CB-1},\kappa_{CB-2},\ldots ,\kappa_{CB-u\;}\right)=\left(\begin{array}{c} 1-\log_{ϜℲ}\left(1+{\prod_{\varepsilon =1}^{u}\left({ϜℲ}^{1-{{}^{\circ}C}_{\kappa_{CB-\varepsilon }}^{RP}}-1\right)}^{\frac{W_{vy-1}\left(1+T\left(\kappa_{CB-\varepsilon }\right)\right)}{\sum_{\varepsilon =1}^{.u}W_{vy-1}\left(1+T\left(\kappa_{CB-\varepsilon }\right)\right)}}\right)\\ +℩\left(1-\log_{ϜℲ}\left(1+{\prod_{\varepsilon =1}^{u}\left({ϜℲ}^{1-{{}^{\circ}C}_{\kappa_{CB-\varepsilon }}^{℩P}}-1\right)}^{\frac{W_{vy-1}\left(1+T\left(\kappa_{CB-\varepsilon }\right)\right)}{\sum_{\varepsilon =1}^{.u}W_{vy-1}\left(1+T\left(\kappa_{CB-\varepsilon }\right)\right)}}\right)\right),\\ -\left(\log_{ϜℲ}\left(1+\prod_{\varepsilon =1}^{u}{\left({ϜℲ}^{-{{}^{\circ}C}_{\kappa_{CB-\varepsilon }}^{RN}}-1\right)}^{\frac{W_{vy-1}\left(1+T\left(\kappa_{CB-\varepsilon }\right)\right)}{\sum_{\varepsilon =1}^{.u}W_{vy-1}\left(1+T\left(\kappa_{CB-\varepsilon }\right)\right)}}\right)\right)\\ +℩\left(-\left(\log_{ϜℲ}\left(1+\prod_{\varepsilon =1}^{u}{\left({ϜℲ}^{-{{}^{\circ}C}_{\kappa_{CB-\varepsilon }}^{℩N}}-1\right)}^{\frac{W_{vy-1}\left(1+T\left(\kappa_{CB-\varepsilon }\right)\right)}{\sum_{\varepsilon =1}^{.u}W_{vy-1}\left(1+T\left(\kappa_{CB-\varepsilon }\right)\right)}}\right)\right)\right),\\ 1-\log_{ϜℲ}\left(1+\prod_{\varepsilon =1}^{u}{\left({ϜℲ}^{1-\xi_{\kappa_{CB-\varepsilon }}^{RP}}-1\right)}^{\frac{W_{vy-1}\left(1+T\left(\kappa_{CB-\varepsilon }\right)\right)}{\sum_{\varepsilon =1}^{.u}W_{vy-1}\left(1+T\left(\kappa_{CB-\varepsilon }\right)\right)}}\right)\\ +℩\left(1-\log_{ϜℲ}\left(1+\prod_{\varepsilon =1}^{u}{\left({ϜℲ}^{1-\xi_{\kappa_{CB-\varepsilon }}^{℩P}}-1\right)}^{\frac{W_{vy-1}\left(1+T\left(\kappa_{CB-\varepsilon }\right)\right)}{\sum_{\varepsilon =1}^{.u}W_{vy-1}\left(1+T\left(\kappa_{CB-\varepsilon }\right)\right)}}\right)\right) \end{array}\right)$$

$${Cir-BCFFPWA}_{t}\left(\kappa_{CB-1},\kappa_{CB-2},\ldots ,\kappa_{CB-u\;}\right)=\left(\begin{array}{c} 1-\log_{ϜℲ}\left(1+{\prod_{\varepsilon =1}^{u}\left({ϜℲ}^{1-{{}^{\circ}C}_{\kappa_{CB-\varepsilon }}^{RP}}-1\right)}^{\frac{W_{vy-1}\left(1+T\left(\kappa_{CB-\varepsilon }\right)\right)}{\sum_{\varepsilon =1}^{.u}W_{vy-1}\left(1+T\left(\kappa_{CB-\varepsilon }\right)\right)}}\right)\\ +℩\left(1-\log_{ϜℲ}\left(1+\prod_{\varepsilon =1}^{u}{\left({ϜℲ}^{1-{{}^{\circ}C}_{\kappa_{CB-\varepsilon }}^{℩P}}-1\right)}^{\frac{W_{vy-1}\left(1+T\left(\kappa_{CB-\varepsilon }\right)\right)}{\sum_{\varepsilon =1}^{.u}W_{vy-1}\left(1+T\left(\kappa_{CB-\varepsilon }\right)\right)}}\right)\right),\\ -\left(\log_{ϜℲ}\left(1+\prod_{\varepsilon =1}^{u}{\left({ϜℲ}^{-{{}^{\circ}C}_{\kappa_{CB-\varepsilon }}^{RN}}-1\right)}^{\frac{W_{vy-1}\left(1+T\left(\kappa_{CB-\varepsilon }\right)\right)}{\sum_{\varepsilon =1}^{.u}W_{vy-1}\left(1+T\left(\kappa_{CB-\varepsilon }\right)\right)}}\right)\right)\\ +℩\left(-\left(\log_{ϜℲ}\left(1+\prod_{\varepsilon =1}^{u}{\left({ϜℲ}^{-{{}^{\circ}C}_{\kappa_{CB-\varepsilon }}^{℩N}}-1\right)}^{\frac{W_{vy-1}\left(1+T\left(\kappa_{CB-\varepsilon }\right)\right)}{\sum_{\varepsilon =1}^{.u}W_{vy-1}\left(1+T\left(\kappa_{CB-\varepsilon }\right)\right)}}\right)\right)\right),\\ \left(\log_{ϜℲ}\left(1+\prod_{\varepsilon =1}^{u}{\left({ϜℲ}^{\xi_{\kappa_{CB-\varepsilon }}^{RP}}-1\right)}^{\frac{W_{vy-1}\left(1+T\left(\kappa_{CB-\varepsilon }\right)\right)}{\sum_{\varepsilon =1}^{.u}W_{vy-1}\left(1+T\left(\kappa_{CB-\varepsilon }\right)\right)}}\right)\right)\\ +℩\left(\log_{ϜℲ}\left(1+\prod_{\varepsilon =1}^{u}{\left({ϜℲ}^{\xi_{\kappa_{CB-\varepsilon }}^{℩P}}-1\right)}^{\frac{W_{vy-1}\left(1+T\left(\kappa_{CB-\varepsilon }\right)\right)}{\sum_{\varepsilon =1}^{.u}W_{vy-1}\left(1+T\left(\kappa_{CB-\varepsilon }\right)\right)}}\right)\right) \end{array}\right)$$

Proof *Omitted*

Property 4 *Idempotency*: ∀ε*if*$\kappa_{CB-\varepsilon }=\kappa_{CB}$, *then*

$${Cir-BCFFPWA}_{tc}\left(\kappa_{CB-1},\kappa_{CB-2},\ldots ,\kappa_{CB-\ddot{u}}\right)=\kappa_{CB}$$

$${Cir-BCFFPWA}_{t}\left(\kappa_{CB-1},\kappa_{CB-2},\ldots ,\kappa_{CB-\ddot{u}}\right)=\kappa_{CB}$$

Property 5 *Boundedness*: *Consider that*$\kappa_{CB}^{-}=\left(\min_{\varepsilon }\left({{}^{\circ}C}_{\kappa_{CB-\varepsilon }}^{RP}\right)+℩\min_{\varepsilon }\left({{}^{\circ}C}_{\kappa_{CB-\varepsilon }}^{℩P}\right),\max_{\varepsilon }\left({{}^{\circ}C}_{\kappa_{CB-\varepsilon }}^{RN}\right)+℩\max_{\varepsilon }\left({{}^{\circ}C}_{\kappa_{CB-\varepsilon }}^{℩N}\right),\min_{\varepsilon }\left(\xi_{\kappa_{CB-\varepsilon }}^{RP}\right)+℩\min_{\varepsilon }\left(\xi_{\kappa_{CB-\varepsilon }}^{℩P}\right)\right)$*and*$\kappa_{CB}^{+}=\left(\max_{\varepsilon }\left({{}^{\circ}C}_{\kappa_{CB-\varepsilon }}^{RP}\right)+℩\max_{\varepsilon }\left({{}^{\circ}C}_{\kappa_{CB-\varepsilon }}^{℩P}\right),\min_{\varepsilon }\left({{}^{\circ}C}_{\kappa_{CB-\varepsilon }}^{RN}\right)+℩\min_{\varepsilon }\left({{}^{\circ}C}_{\kappa_{CB-\varepsilon }}^{℩N}\right),\max_{\varepsilon }\left(\xi_{\kappa_{CB-\varepsilon }}^{RP}\right)+℩\max_{\varepsilon }\left(\xi_{\kappa_{CB-\varepsilon }}^{℩P}\right)\right)$*for t-conorm and similarly for t-norm*, *then*

$$\kappa_{CB}^{-}\leq {Cir-BCFFPWA}_{tc}\left(\kappa_{CB-1},\kappa_{CB-2},\ldots ,\kappa_{CB-\ddot{u}}\right)\leq \kappa_{CB}^{+}$$

$$\kappa_{CB}^{-}\leq {Cir-BCFFPWA}_{t}\left(\kappa_{CB-1},\kappa_{CB-2},\ldots ,\kappa_{CB-\ddot{u}}\right)\leq \kappa_{CB}^{+}$$

Property 6 *Monotonicity*: ∀ε*if*$\kappa_{CB-\varepsilon }\leq K_{CB}^{/}$, *then*

$${Cir-BCFFWA}_{tc}\left(\kappa_{CB-1},\kappa_{CB-2},\ldots ,\kappa_{CB-\ddot{u}}\right)\leq {Cir-BCFFWA}_{tc}\left(K_{CB-1}^{/},K_{CB-2}^{/},\ldots ,K_{CB-\ddot{u}}^{/}\right)$$

$${Cir-BCFFWA}_{t}\left(\kappa_{CB-1},\kappa_{CB-2},\ldots ,\kappa_{CB-\ddot{u}}\right)\leq {Cir-BCFFWA}_{t}\left(K_{CB-1}^{/},K_{CB-2}^{/},\ldots ,K_{CB-\ddot{u}}^{/}\right)$$

Definition 11 *Let's consider Cir-BCFNs i*.*e*. $\kappa_{CB-\varepsilon }=\left({{}^{\circ}C}_{\kappa_{CB-\varepsilon }}^{P},{{}^{\circ}C}_{\kappa_{CB-\varepsilon }}^{N},\xi_{\kappa_{CB-\varepsilon }}^{P}\right)=\left({{}^{\circ}C}_{\kappa_{CB-\varepsilon }}^{RP}+℩{{}^{\circ}C}_{\kappa_{CB-\varepsilon }}^{℩P},{{}^{\circ}C}_{\kappa_{CB-\varepsilon }}^{RN}+℩{{}^{\circ}C}_{\kappa_{CB-\varepsilon }}^{℩N},\xi_{\kappa_{CB}-\varepsilon }^{RP}+℩\xi_{\kappa_{CB-\varepsilon }}^{℩P}\right)$, ε=1,2,3,…,u, *then*

$${Cir-BCFFPG}_{tc}\left(\kappa_{CB-1},\kappa_{CB-2},\ldots ,\kappa_{CB-u\;}\right)={{⊗}_{\varepsilon =1}^{u}}_{tc}{\kappa_{CB-\varepsilon }}^{\frac{\left(1+T\left(\kappa_{CB-\varepsilon }\right)\right)}{\sum_{\varepsilon =1}^{.u}\left(1+T\left(\kappa_{CB-\varepsilon }\right)\right)}}$$

$${Cir-BCFFPG}_{t}\left(\kappa_{CB-1},\kappa_{CB-2},\ldots ,\kappa_{CB-u\;}\right)={{⊗}_{\varepsilon =1}^{u}}_{t}{\kappa_{CB-\varepsilon }}^{\frac{\left(1+T\left(\kappa_{CB-\varepsilon }\right)\right)}{\sum_{\varepsilon =1}^{.\ddot{u}}\left(1+T\left(\kappa_{CB-\varepsilon }\right)\right)}}$$

*is defined as Cir-BCFFFPG operator and*$T\left(\kappa_{CB-\varepsilon }\right)=\sum_{\begin{array}{c} i=1\\ i\neq \varepsilon \end{array}}^{u}\;\mathrm{Sup}\left(\kappa_{CB-\varepsilon },\kappa_{CB-i}\right)$.

Theorem 3 *Consider two sets of Cir-BCFNs i*.*e*. $\kappa_{CB-\varepsilon }=\left({{}^{\circ}C}_{\kappa_{CB-\varepsilon }}^{P},{{}^{\circ}C}_{\kappa_{CB-\varepsilon }}^{N},\xi_{\kappa_{CB-\varepsilon }}^{P}\right)=\left({{}^{\circ}C}_{\kappa_{CB-\varepsilon }}^{RP}+℩{{}^{\circ}C}_{\kappa_{CB-\varepsilon }}^{℩P},{{}^{\circ}C}_{\kappa_{CB-\varepsilon }}^{RN}+℩{{}^{\circ}C}_{\kappa_{CB-\varepsilon }}^{℩N},\xi_{\kappa_{CB-\varepsilon }}^{RP}+℩\xi_{\kappa_{CB-\varepsilon }}^{℩P}\right)$, ε=1,2,3…,u*then by employing the above Equation*, *the aggregated value would be a Cir-BCFN and*

$${Cir-BCFFPG}_{tc}\left(\kappa_{CB-1},\kappa_{CB-2},\ldots ,\kappa_{CB-u\;}\right)=\left(\begin{array}{c} \log_{ϜℲ}\left(1+{\prod_{\varepsilon =1}^{u}\left({ϜℲ}^{{{}^{\circ}C}_{\kappa_{CB-\varepsilon }}^{RP}}-1\right)}^{\frac{\left(1+T\left(\kappa_{CB-\varepsilon }\right)\right)}{\sum_{\varepsilon =1}^{.\ddot{u}}\left(1+T\left(\kappa_{CB-\varepsilon }\right)\right)}}\right)\\ +℩\left(\log_{ϜℲ}\left(1+\prod_{\varepsilon =1}^{u}{\left({ϜℲ}^{{{}^{\circ}C}_{\kappa_{CB-\varepsilon }}^{℩P}}-1\right)}^{\frac{\left(1+T\left(\kappa_{CB-\varepsilon }\right)\right)}{\sum_{\varepsilon =1}^{.\ddot{u}}\left(1+T\left(\kappa_{CB-\varepsilon }\right)\right)}}\right)\right),\\ -1+\log_{ϜℲ}\left(1+{\prod_{\varepsilon =1}^{u}\left({ϜℲ}^{1+{{}^{\circ}C}_{\kappa_{CB-\varepsilon }}^{RN}}-1\right)}^{\frac{\left(1+T\left(\kappa_{CB-\varepsilon }\right)\right)}{\sum_{\varepsilon =1}^{.u}\left(1+T\left(\kappa_{CB-\varepsilon }\right)\right)}}\right)\\ +℩\left(-1+\log_{ϜℲ}\left(1+\prod_{\varepsilon =1}^{u}{\left({ϜℲ}^{1+{{}^{\circ}C}_{\kappa_{CB-\varepsilon }}^{℩N}}-1\right)}^{\frac{\left(1+T\left(\kappa_{CB-\varepsilon }\right)\right)}{\sum_{\varepsilon =1}^{.\ddot{u}}\left(1+T\left(\kappa_{CB-\varepsilon }\right)\right)}}\right)\right),\\ \log_{ϜℲ}\left(1+\prod_{\varepsilon =1}^{u}{\left({ϜℲ}^{\xi_{\kappa_{CB-\varepsilon }}^{RP}}-1\right)}^{\frac{\left(1+T\left(\kappa_{CB-\varepsilon }\right)\right)}{\sum_{\varepsilon =1}^{.\ddot{u}}\left(1+T\left(\kappa_{CB-\varepsilon }\right)\right)}}\right)\\ +℩\left(\log_{ϜℲ}\left(1+\prod_{\varepsilon =1}^{u}{\left({ϜℲ}^{\xi_{\kappa_{CB-\varepsilon }}^{℩P}}-1\right)}^{\frac{\left(1+T\left(\kappa_{CB-\varepsilon }\right)\right)}{\sum_{\varepsilon =1}^{.\ddot{u}}\left(1+T\left(\kappa_{CB-\varepsilon }\right)\right)}}\right)\right) \end{array}\right)$$

$${Cir-BCFFPG}_{t}\left(\kappa_{CB-1},\kappa_{CB-2},\ldots ,\kappa_{CB-u\;}\right)=\left(\begin{array}{c} \log_{ϜℲ}\left(1+{\prod_{\varepsilon =1}^{u}\left({ϜℲ}^{{{}^{\circ}C}_{\kappa_{CB-\varepsilon }}^{RP}}-1\right)}^{\frac{\left(1+T\left(\kappa_{CB-\varepsilon }\right)\right)}{\sum_{\varepsilon =1}^{.u}\left(1+T\left(\kappa_{CB-\varepsilon }\right)\right)}}\right)\\ +℩\left(\log_{ϜℲ}\left(1+{\prod_{\varepsilon =1}^{u}\left({ϜℲ}^{{{}^{\circ}C}_{\kappa_{CB-\varepsilon }}^{℩P}}-1\right)}^{\frac{\left(1+T\left(\kappa_{CB-\varepsilon }\right)\right)}{\sum_{\varepsilon =1}^{.u}\left(1+T\left(\kappa_{CB-\varepsilon }\right)\right)}}\right)\right),\\ -1+\log_{ϜℲ}\left(1+\prod_{\varepsilon =1}^{u}{\left({ϜℲ}^{1+{{}^{\circ}C}_{\kappa_{CB-\varepsilon }}^{RN}}-1\right)}^{\frac{\left(1+T\left(\kappa_{CB-\varepsilon }\right)\right)}{\sum_{\varepsilon =1}^{.\ddot{u}}\left(1+T\left(\kappa_{CB-\varepsilon }\right)\right)}}\right)\\ +℩\left(-1+\log_{ϜℲ}\left(1+{\prod_{\varepsilon =1}^{u}\left({ϜℲ}^{1+{{}^{\circ}C}_{\kappa_{CB-\varepsilon }}^{RN}}-1\right)}^{\frac{\left(1+T\left(\kappa_{CB-\varepsilon }\right)\right)}{\sum_{\varepsilon =1}^{.\ddot{u}}\left(1+T\left(\kappa_{CB-\varepsilon }\right)\right)}}\right)\right),\\ 1-\log_{ϜℲ}\left(1+\prod_{\varepsilon =1}^{u}{\left({ϜℲ}^{1-\xi_{\kappa_{CB-\varepsilon }}^{RP}}-1\right)}^{\frac{\left(1+T\left(\kappa_{CB-\varepsilon }\right)\right)}{\sum_{\varepsilon =1}^{.u}\left(1+T\left(\kappa_{CB-\varepsilon }\right)\right)}}\right)\\ +℩\left(1-\log_{ϜℲ}\left(1+{\prod_{\varepsilon =1}^{u}\left({ϜℲ}^{1-\xi_{\kappa_{CB-\varepsilon }}^{℩P}}-1\right)}^{\frac{\left(1+T\left(\kappa_{CB-\varepsilon }\right)\right)}{\sum_{\varepsilon =1}^{.u}\left(1+T\left(\kappa_{CB-\varepsilon }\right)\right)}}\right)\right) \end{array}\right)$$

Proof *Let*

$${Cir-BCFFPG}_{tc}\left(\kappa_{CB-1}\right)⊗{Cir-BCFFPG}_{tc}\left(\kappa_{CB-2}\right)=\left(\begin{array}{c} \begin{array}{c} \log_{ϜℲ}\left(+\frac{{\left({ϜℲ}^{{{}^{\circ}C}_{\kappa_{CB-\varepsilon }}^{RP}}-1\right)}^{A_{1}}}{{\left(ϜℲ-1\right)}^{A_{D+1}-1}}\right)+℩\left(\log_{ϜℲ}\left(1+\frac{{\left({ϜℲ}^{{{}^{\circ}C}_{BCBFS-1}^{℩P}}-1\right)}^{A_{1}}}{{\left(ϜℲ-1\right)}^{A_{D+1}-1}}\right)\right),-1+\log_{ϜℲ}\left(1+\frac{{\left({ϜℲ}^{1+{{}^{\circ}C}_{\kappa_{CB-\varepsilon }}^{℩N}}-1\right)}^{A_{1}}}{{\left(ϜℲ-1\right)}^{A_{1}-1}}\right)+℩\left(-1+\left(\log_{ϜℲ}\left(\begin{array}{c} 1+\\ \frac{{\left({ϜℲ}^{1+{{}^{\circ}C}_{\kappa_{CB-\varepsilon }}^{℩N}}-1\right)}^{A_{1}}}{{\left(ϜℲ-1\right)}^{A_{1}-1}} \end{array}\right)\right)\right),\\ \log_{ϜℲ}\left(1+\frac{{\left(\xi^{\xi_{\kappa_{CB-\varepsilon }}^{RP}}-1\right)}^{A_{1}}}{{\left(ϜℲ-1\right)}^{A_{1}-1}}\right) \end{array}\\ +℩\left(\log_{ϜℲ}\left(1+\frac{{\left(\xi^{\xi_{\kappa_{CB-\varepsilon }}^{℩P}}-1\right)}^{A_{1}}}{{\left(ϜℲ-1\right)}^{A_{1}-1}}\right)\right) \end{array}\right)⊗\left(\begin{array}{c} \begin{array}{c} \log_{ϜℲ}\left(1+\frac{{\left({ϜℲ}^{{{}^{\circ}C}_{\kappa_{CB-\varepsilon }}^{RP}}-1\right)}^{A_{2}}}{{\left(ϜℲ-1\right)}^{A_{2}-1}}\right)+℩\left(\log_{ϜℲ}\left(1+\frac{{\left({ϜℲ}^{{{}^{\circ}C}_{\kappa_{CB-\varepsilon }}^{℩P}}-1\right)}^{A_{2}}}{{\left(ϜℲ-1\right)}^{A_{2}-1}}\right)\right),-1+\log_{ϜℲ}\left(1+\frac{{\left({ϜℲ}^{1+{{}^{\circ}C}_{\kappa_{CB-\varepsilon }}^{℩N}}-1\right)}^{A_{2}}}{{\left(ϜℲ-1\right)}^{A_{2}-1}}\right)+℩\left(-1+\left(\log_{ϜℲ}\left(1+\frac{{\left({ϜℲ}^{1+{{}^{\circ}C}_{\kappa_{CB-\varepsilon }}^{℩N}}-1\right)}^{A_{2}}}{{\left(ϜℲ-1\right)}^{A_{2}-1}}\right)\right)\right),\\ \log_{ϜℲ}\left(1+\frac{{\left({ϜℲ}^{\xi_{\kappa_{CB-\varepsilon }}^{RP}}-1\right)}^{A_{2}}}{{\left(ϜℲ-1\right)}^{A_{2}-1}}\right) \end{array}\\ +℩\left(\log_{ϜℲ}\left(1+\frac{{\left(\xi^{\xi_{\kappa_{CB-\varepsilon }}^{℩P}}-1\right)}^{A_{2}}}{{\left(ϜℲ-1\right)}^{A_{2}-1}}\right)\right) \end{array}\right)$$

$${Cir-BCir-FFPG}_{tc}\left(\kappa_{CB-1}\right)⊗{CBCFFPG}_{tc}\left(\kappa_{CB-2}\right)=\left(\begin{array}{c} \log_{ϜℲ}\left(1+\prod_{\varepsilon =1}^{2}{\left({ϜℲ}^{{{}^{\circ}C}_{\kappa_{CB-\varepsilon }}^{RP}}-1\right)}^{A_{\varepsilon }}\right)\\ +℩\left(\log_{ϜℲ}\left(1+\prod_{\varepsilon =1}^{2}{\left({ϜℲ}^{{{}^{\circ}C}_{\kappa_{CB-\varepsilon }}^{℩P}}-1\right)}^{A_{\varepsilon }}\right)\right),\\ -1+\left(\log_{ϜℲ}\left(1+\prod_{\varepsilon =1}^{2}{\left({ϜℲ}^{1+{{}^{\circ}C}_{\kappa_{CB-\varepsilon }}^{RN}}-1\right)}^{A_{\varepsilon }}\right)\right)\\ +℩\left(-1+\left(\log_{ϜℲ}\left(1+\prod_{\varepsilon =1}^{2}{\left({ϜℲ}^{1+{{}^{\circ}C}_{\kappa_{CB-\varepsilon }}^{℩N}}-1\right)}^{A_{\varepsilon }}\right)\right)\right),\\ \log_{ϜℲ}\left(1+\prod_{\varepsilon =1}^{2}{\left({ϜℲ}^{\xi_{\kappa_{CB-\varepsilon }}^{RP}}-1\right)}^{A_{\varepsilon }}\right)\\ +℩\left(\log_{ϜℲ}\left(1+\prod_{\varepsilon =1}^{2}{\left({ϜℲ}^{\xi_{\kappa_{CB-\varepsilon }}^{℩P}}-1\right)}^{A_{\varepsilon }}\right)\right) \end{array}\right)$$

*Suppose the given equation holds for*u=D.

$${Cir-BCFFPG}_{tc}\left(\kappa_{CB-1},\kappa_{CB-2},\ldots ,\kappa_{CB-D}\right)=\left(\begin{array}{c} \log_{ϜℲ}\left(1+\prod_{\varepsilon =1}^{D}{\left({ϜℲ}^{{{}^{\circ}C}_{\kappa_{CB-\varepsilon }}^{RP}}-1\right)}^{A_{\varepsilon }}\right)\\ +℩\left(\log_{ϜℲ}\left(1+{{{}^{\circ}C}_{\varepsilon =1}^{D}\left({ϜℲ}^{{{}^{\circ}C}_{\kappa_{CB-\varepsilon }}^{℩P}}-1\right)}^{A_{\varepsilon }}\right)\right),\\ -1+\left(\log_{ϜℲ}\left(1+\prod_{\varepsilon =1}^{D}{\left({ϜℲ}^{1+{{}^{\circ}C}_{\kappa_{CB-\varepsilon }}^{RN}}-1\right)}^{A_{\varepsilon }}\right)\right)\\ +℩\left(-1+\left(\log_{ϜℲ}\left(1+\prod_{\varepsilon =1}^{D}{\left({ϜℲ}^{1+{{}^{\circ}C}_{\kappa_{CB-\varepsilon }}^{℩N}}-1\right)}^{A_{\varepsilon }}\right)\right)\right),\\ \log_{ϜℲ}\left(1+\prod_{\varepsilon =1}^{D}{\left({ϜℲ}^{\xi_{\kappa_{CB-\varepsilon }}^{RP}}-1\right)}^{A_{\varepsilon }}\right)\\ +℩\left(\log_{ϜℲ}\left(1+\prod_{\varepsilon =1}^{D}{\left({ϜℲ}^{\xi_{\kappa_{CB-\varepsilon }}^{℩P}}-1\right)}^{A_{\varepsilon }}\right)\right) \end{array}\right)$$

*Now we will prove that the given equation is true for*u=D+1.

$${Cir-BCFFPG}_{tc}\left(\kappa_{CB-1},\kappa_{CB-2},\ldots ,\kappa_{CB-\left(D+1\right)}\right)=\left(\begin{array}{c} \log_{ϜℲ}\left(1+\prod_{\varepsilon =1}^{D}{\left({ϜℲ}^{{{}^{\circ}C}_{\kappa_{CB-\varepsilon }}^{RP}}-1\right)}^{A_{\varepsilon }}\right)\\ +℩\left(\log_{ϜℲ}\left(1+\prod_{\varepsilon =1}^{D}{\left({ϜℲ}^{{{}^{\circ}C}_{\kappa_{CB-\varepsilon }}^{℩P}}-1\right)}^{A_{\varepsilon }}\right)\right),\\ -1+\left(\log_{ϜℲ}\left(1+\prod_{\varepsilon =1}^{D}{\left({ϜℲ}^{1+{{}^{\circ}C}_{\kappa_{CB-\varepsilon }}^{RN}}-1\right)}^{A_{\varepsilon }}\right)\right)\\ +℩\left(-1+\left(\log_{ϜℲ}\left(1+\prod_{\varepsilon =1}^{D}{\left({ϜℲ}^{1+{{}^{\circ}C}_{\kappa_{CB-\varepsilon }}^{℩N}}-1\right)}^{A_{\varepsilon }}\right)\right)\right),\\ \log_{ϜℲ}\left(1+\prod_{\varepsilon =1}^{D}{\left({ϜℲ}^{\xi_{\kappa_{CB-\varepsilon }}^{RP}}-1\right)}^{A_{\varepsilon }}\right)\\ +℩\left(\log_{ϜℲ}\left(1+\prod_{\varepsilon =1}^{D}{\left({ϜℲ}^{\xi_{\kappa_{CB-\varepsilon }}^{℩P}}-1\right)}^{A_{\varepsilon }}\right)\right) \end{array}\right)⊗\left(\begin{array}{c} \begin{array}{c} \log_{ϜℲ}\left(1+\frac{{\left({ϜℲ}^{{{}^{\circ}C}_{\kappa_{CB-\left(D+1\right)}}^{RP}}-1\right)}^{A_{D+1}}}{{\left(ϜℲ-1\right)}^{A_{D+1}-1}}\right)+℩\left(\log_{ϜℲ}\left(1+\frac{{\left({ϜℲ}^{{{}^{\circ}C}_{\kappa_{CB-\left(D+1\right)}}^{℩P}}-1\right)}^{A_{D+1}}}{{\left(ϜℲ-1\right)}^{A_{D+1}-1}}\right)\right),-1+\log_{ϜℲ}\left(1+\frac{{\left({ϜℲ}^{1+{{}^{\circ}C}_{\kappa_{CB-\left(D+1\right)}}^{RN}}-1\right)}^{A_{D+1}}}{{\left(ϜℲ-1\right)}^{A_{D+1}-1}}\right)+℩\left(-1+\left(\log_{ϜℲ}\left(1+\frac{{\left({ϜℲ}^{1+{{}^{\circ}C}_{\kappa_{CB-\left(D+1\right)}}^{℩N}}-1\right)}^{A_{D+1}}}{{\left(ϜℲ-1\right)}^{A_{D+1}-1}}\right)\right)\right),\\ \log_{ϜℲ}\left(1+\frac{{\left({ϜℲ}^{\xi_{\kappa_{CB-\left(D+1\right)}}^{RP}}-1\right)}^{A_{D+1}}}{{\left(ϜℲ-1\right)}^{A_{D+1}-1}}\right) \end{array}\\ +℩\left(\log_{ϜℲ}\left(1+\frac{{\left({ϜℲ}^{\xi_{\kappa_{CB-\left(D+1\right)}}^{℩P}}-1\right)}^{A_{D+1}}}{{\left(ϜℲ-1\right)}^{A_{D+1}-1}}\right)\right) \end{array}\right)$$

$${Cir-BCFFPG}_{tc}\left(\kappa_{CB-1},\kappa_{CB-2},\ldots ,\kappa_{CB-\left(D+1\right)}\right)=\left(\begin{array}{c} \log_{ϜℲ}\left(1+\frac{{\prod_{\varepsilon =1}^{D}\left({ϜℲ}^{{{}^{\circ}C}_{\kappa_{CB-\varepsilon }}^{RP}}-1\right)}^{A_{\varepsilon }}}{{\left(ϜℲ-1\right)}^{\sum_{\varepsilon =1}^{D+1}A_{\varepsilon }-1}}\right)\\ +℩\left(\log_{ϜℲ}\left(1+\frac{{\prod_{\varepsilon =1}^{D}\left({ϜℲ}^{{{}^{\circ}C}_{\kappa_{CB-\varepsilon }}^{℩P}}-1\right)}^{A_{\varepsilon }}}{{\left(ϜℲ-1\right)}^{\sum_{\varepsilon =1}^{D+1}A_{\varepsilon }-1}}\right)\right),\\ -1+\log_{ϜℲ}\left(1+\frac{{\prod_{\varepsilon =1}^{D}\left({ϜℲ}^{1+{{}^{\circ}C}_{\kappa_{CB-\varepsilon }}^{℩N}}-1\right)}^{A_{\varepsilon }}}{{\left(ϜℲ-1\right)}^{\sum_{\varepsilon =1}^{D+1}A_{\varepsilon }-1}}\right)\\ +℩\left(-1+\log_{ϜℲ}\left(1+\frac{\prod_{\varepsilon =1}^{D}{\left({ϜℲ}^{1+{{}^{\circ}C}_{\kappa_{CB-\varepsilon }}^{℩N}}-1\right)}^{A_{\varepsilon }}}{{\left(ϜℲ-1\right)}^{\sum_{\varepsilon =1}^{D+1}A_{\varepsilon }-1}}\right)\right),\\ \log_{ϜℲ}\left(1+\frac{{\prod_{\varepsilon =1}^{D}\left({ϜℲ}^{\xi_{\kappa_{CB-\varepsilon }}^{RP}}-1\right)}^{A_{\varepsilon }}}{{\left(ϜℲ-1\right)}^{\sum_{\varepsilon =1}^{D+1}A_{\varepsilon }-1}}\right)\\ +℩\left(\log_{ϜℲ}\left(1+\frac{{\prod_{\varepsilon =1}^{D}\left({ϜℲ}^{\xi_{\kappa_{CB-\varepsilon }}^{℩P}}-1\right)}^{A_{\varepsilon }}}{{\left(ϜℲ-1\right)}^{\sum_{\varepsilon =1}^{D+1}A_{\varepsilon }-1}}\right)\right) \end{array}\right)⊗\left(\begin{array}{c} \begin{array}{c} \log_{ϜℲ}\left(1+\frac{{\left({ϜℲ}^{{{}^{\circ}C}_{\kappa_{CB-\left(D+1\right)}}^{RP}}-1\right)}^{A_{D+1}}}{{\left(ϜℲ-1\right)}^{A_{D+1}-1}}\right)+℩\left(\log_{ϜℲ}\left(1+\frac{{\left({ϜℲ}^{{{}^{\circ}C}_{\kappa_{CB-\left(D+1\right)}}^{℩P}}-1\right)}^{A_{D+1}}}{{\left(ϜℲ-1\right)}^{A_{D+1}-1}}\right)\right),-1+\log_{ϜℲ}\left(1+\frac{{\left({ϜℲ}^{1+{{}^{\circ}C}_{\kappa_{CB-\left(D+1\right)}}^{RN}}-1\right)}^{A_{D+1}}}{{\left(ϜℲ-1\right)}^{A_{D+1}-1}}\right)+℩\left(-1+\left(\log_{ϜℲ}\left(1+\frac{{\left({ϜℲ}^{1+{{}^{\circ}C}_{\kappa_{CB-\left(D+1\right)}}^{℩N}}-1\right)}^{A_{D+1}}}{{\left(ϜℲ-1\right)}^{A_{D+1}-1}}\right)\right)\right),\\ \log_{ϜℲ}\left(1+\frac{{\left({ϜℲ}^{\xi_{\kappa_{CB-\left(D+1\right)}}^{RP}}-1\right)}^{A_{D+1}}}{{\left(ϜℲ-1\right)}^{A_{D+1}-1}}\right) \end{array}\\ +℩\left(\log_{ϜℲ}\left(1+\frac{{\left({ϜℲ}^{\xi_{\kappa_{CB-\left(D+1\right)}}^{℩P}}-1\right)}^{A_{D+1}}}{{\left(ϜℲ-1\right)}^{A_{D+1}-1}}\right)\right) \end{array}\right)$$

$$=\left(\begin{array}{c} \log_{ϜℲ}\left(1+\prod_{\varepsilon =1}^{D+1}{\left({ϜℲ}^{{{}^{\circ}C}_{\kappa_{CB-\varepsilon }}^{RP}}-1\right)}^{A_{\varepsilon }}\right)\\ +℩\left(\log_{ϜℲ}\left(1+\prod_{\varepsilon =1}^{D+1}{\left({ϜℲ}^{{{}^{\circ}C}_{\kappa_{CB-\varepsilon }}^{℩P}}-1\right)}^{A_{\varepsilon }}\right)\right),\\ -1+\left(\log_{ϜℲ}\left(1+\prod_{\varepsilon =1}^{D+1}{\left({ϜℲ}^{1+{{}^{\circ}C}_{\kappa_{CB-\varepsilon }}^{RN}}-1\right)}^{A_{\varepsilon }}\right)\right)\\ +℩\left(-1+\left(\log_{ϜℲ}\left(1+\prod_{\varepsilon =1}^{D+1}{\left({ϜℲ}^{1+{{}^{\circ}C}_{\kappa_{CB-\varepsilon }}^{℩N}}-1\right)}^{A_{\varepsilon }}\right)\right)\right),\\ \log_{ϜℲ}\left(1+\prod_{\varepsilon =1}^{D+1}{\left({ϜℲ}^{\xi_{\kappa_{CB-\varepsilon }}^{RP}}-1\right)}^{A_{\varepsilon }}\right)\\ +℩\left(\log_{ϜℲ}\left(1+\prod_{\varepsilon =1}^{D+1}{\left({ϜℲ}^{\xi_{\kappa_{CB-\varepsilon }}^{℩P}}-1\right)}^{A_{\varepsilon }}\right)\right) \end{array}\right)$$

*So*, *this implies that this equation holds for*u=D+1,*So the result is true for all u*.

*Consider two sets of Cir-BCFNs i*.*e*. $\kappa_{CB-\varepsilon }=\left({{}^{\circ}C}_{\kappa_{CB-\varepsilon }}^{P},{{}^{\circ}C}_{\kappa_{CB-\varepsilon }}^{N},\xi_{\kappa_{CB-\varepsilon }}^{P}\right)=\left({{}^{\circ}C}_{\kappa_{CB-\varepsilon }}^{RP}+℩{{}^{\circ}C}_{\kappa_{CB-\varepsilon }}^{℩P},{{}^{\circ}C}_{\kappa_{CB-\varepsilon }}^{RN}+℩{{}^{\circ}C}_{\kappa_{CB-\varepsilon }}^{℩N},\xi_{\kappa_{CB-\varepsilon }}^{RP}+℩\xi_{\kappa_{CB-\varepsilon }}^{℩P}\right)$*and*$\kappa_{CB-\varepsilon }^{/}=\left({{}^{\circ}C}_{\kappa_{CB-\varepsilon }}^{P},{{}^{\circ}C}_{\kappa_{CB-\varepsilon }}^{N},\xi_{\kappa_{CB-\varepsilon }}^{P}\right)=\left({{}^{\circ}C}_{\kappa_{CB-\varepsilon }}^{RP}+℩{{}^{\circ}C}_{\kappa_{CB-\varepsilon }}^{℩P},{{}^{\circ}C}_{\kappa_{CB-\varepsilon }}^{RN}+℩{{}^{\circ}C}_{\kappa_{CB-\varepsilon }}^{℩N},\xi_{\kappa_{CB-\varepsilon }}^{RP}+℩\xi_{\kappa_{CB-\varepsilon }}^{℩P}\right)$*then Cir-BFFPG operator has underneath properties*.

Property 7 *Idempotency*: ∀ε*if*$\kappa_{CB-\varepsilon }=\kappa_{CB}$, *then*

$${Cir-BCFFPG}_{tc}\left(\kappa_{CB-1},\kappa_{CB-2},\ldots ,\kappa_{CB-\ddot{u}}\right)=\kappa_{CB}$$

$${Cir-BCFFPG}_{t}\left(\kappa_{CB-1},\kappa_{CB-2},\ldots ,\kappa_{CB-\ddot{u}}\right)=\kappa_{CB}$$

Property 8 *Boundedness*: *Assume that*$\kappa_{CB}^{-}=\left(\min_{\varepsilon }\left({{}^{\circ}C}_{\kappa_{CB-\varepsilon }}^{RP}\right)+℩\min_{\varepsilon }\left({{}^{\circ}C}_{\kappa_{CB-\varepsilon }}^{℩P}\right),\max_{\varepsilon }\left({{}^{\circ}C}_{\kappa_{CB-\varepsilon }}^{RN}\right)+℩\max_{\varepsilon }\left({{}^{\circ}C}_{\kappa_{CB-\varepsilon }}^{℩N}\right),\min_{\varepsilon }\left(\xi_{\kappa_{CB-\varepsilon }}^{RP}\right)+℩\min_{\varepsilon }\left(\xi_{\kappa_{CB-\varepsilon }}^{℩P}\right)\right)$*and*$\kappa_{CB}^{+}=\left(\max_{\varepsilon }\left({{}^{\circ}C}_{\kappa_{CB-\varepsilon }}^{RP}\right)+℩\max_{\varepsilon }\left({{}^{\circ}C}_{\kappa_{CB-\varepsilon }}^{℩P}\right),\min_{\varepsilon }\left({{}^{\circ}C}_{\kappa_{CB-\varepsilon }}^{RN}\right)+℩\min_{\varepsilon }\left({{}^{\circ}C}_{\kappa_{CB-\varepsilon }}^{℩N}\right),\max_{\varepsilon }\left(\xi_{\kappa_{CB-\varepsilon }}^{RP}\right)+℩\max_{\varepsilon }\left(\xi_{\kappa_{CB-\varepsilon }}^{℩P}\right)\right)$*for t-conorm and similarly for t-norm*, *then*

$$\kappa_{CB}^{-}\leq {Cir-BCFFPG}_{tc}\left(\kappa_{CB-1},\kappa_{CB-2},\ldots ,\kappa_{CB-\ddot{u}}\right)\leq \kappa_{CB}^{+}$$

$$\kappa_{CB}^{-}\leq {Cir-BCFFPG}_{t}\left(\kappa_{CB-1},\kappa_{CB-2},\ldots ,\kappa_{CB-\ddot{u}}\right)\leq \kappa_{CB}^{+}$$

Property 9 *Monotonicity*: ∀ε*if*$\kappa_{CB-\varepsilon }\leq K_{CB}^{/}$, *then*

$${Cir-BCFFPG}_{tc}\left(\kappa_{CB-1},\kappa_{CB-2},\ldots ,\kappa_{CB-\ddot{u}}\right)\leq {Cir-BCFFPG}_{tc}\left(K_{CB-1}^{/},K_{CB-2}^{/},\ldots ,K_{CB-\ddot{u}}^{/}\right)$$

$${Cir-BCFFPG}_{t}\left(\kappa_{CB-1},\kappa_{CB-2},\ldots ,\kappa_{CB-\ddot{u}}\right)\leq {Cir-BCFFPG}_{t}\left(K_{CB-1}^{/},K_{CB-2}^{/},\ldots ,K_{CB-\ddot{u}}^{/}\right)$$

Definition 12 *Let's consider Cir-BCFNs i*.*e*. $\kappa_{CB-\varepsilon }=\left({{}^{\circ}C}_{\kappa_{CB-\varepsilon }}^{P},{{}^{\circ}C}_{\kappa_{CB-\varepsilon }}^{N},\xi_{\kappa_{CB-\varepsilon }}^{P}\right)=\left({{}^{\circ}C}_{\kappa_{CB-\varepsilon }}^{RP}+℩{{}^{\circ}C}_{\kappa_{CB-\varepsilon }}^{℩P},{{}^{\circ}C}_{\kappa_{CB-\varepsilon }}^{RN}+℩{{}^{\circ}C}_{\kappa_{CB-\varepsilon }}^{℩N},\xi_{\kappa_{CB}-\varepsilon }^{RP}+℩\xi_{\kappa_{CB-\varepsilon }}^{℩P}\right)$, ε=1,2,3,…,u, *then*

$${Cir-BCFFPWG}_{tc}\left(\kappa_{CB-1},\kappa_{CB-2},\ldots ,\kappa_{CB-u\;}\right)={{⊗}_{\varepsilon =1}^{u}}_{tc}{\kappa_{CB-\varepsilon }}^{\frac{W_{vy-1}\left(1+T\left(\kappa_{CB-\varepsilon }\right)\right)}{\sum_{\varepsilon =1}^{.u}W_{vy-1}\left(1+T\left(\kappa_{CB-\varepsilon }\right)\right)}}$$

$${Cir-BCFFPWG}_{t}\left(\kappa_{CB-1},\kappa_{CB-2},\ldots ,\kappa_{CB-u\;}\right)={{⊗}_{\varepsilon =1}^{u}}_{t}{\kappa_{CB-\varepsilon }}^{\frac{W_{vy-1}\left(1+T\left(\kappa_{CB-\varepsilon }\right)\right)}{\sum_{\varepsilon =1}^{.\ddot{u}}W_{vy-1}\left(1+T\left(\kappa_{CB-\varepsilon }\right)\right)}}$$

*is defined as Cir-BCFFPWG operator and*$T\left(\kappa_{CB-\varepsilon }\right)=\sum_{\begin{array}{c} i=1\\ i\neq \varepsilon \end{array}}^{u}\;\mathrm{Sup}\left(\kappa_{CB-\varepsilon },\kappa_{CB-i}\right)$.

Theorem 4 *Consider two sets of Cir-BCFNs i*.*e*. $\kappa_{CB-\varepsilon }=\left({{}^{\circ}C}_{\kappa_{CB-\varepsilon }}^{P},{{}^{\circ}C}_{\kappa_{CB-\varepsilon }}^{N},\xi_{\kappa_{CB-\varepsilon }}^{P}\right)=\left({{}^{\circ}C}_{\kappa_{CB-\varepsilon }}^{RP}+℩{{}^{\circ}C}_{\kappa_{CB-\varepsilon }}^{℩P},{{}^{\circ}C}_{\kappa_{CB-\varepsilon }}^{RN}+℩{{}^{\circ}C}_{\kappa_{CB-\varepsilon }}^{℩N},\xi_{\kappa_{CB-\varepsilon }}^{RP}+℩\xi_{\kappa_{CB-\varepsilon }}^{℩P}\right)$, ε=1,2,3…,u*then by employing the above Equation*, *the aggregated value would be a Cir-BCFN and*

$${Cir-BCFFPWG}_{tc}\left(\kappa_{CB-1},\kappa_{CB-2},\ldots ,\kappa_{CB-u\;}\right)=\left(\begin{array}{c} \log_{ϜℲ}\left(1+{\prod_{\varepsilon =1}^{u}\left({ϜℲ}^{{{}^{\circ}C}_{\kappa_{CB-\varepsilon }}^{RP}}-1\right)}^{\frac{W_{vy-1}\left(1+T\left(\kappa_{CB-\varepsilon }\right)\right)}{\sum_{\varepsilon =1}^{.\ddot{u}}W_{vy-1}\left(1+T\left(\kappa_{CB-\varepsilon }\right)\right)}}\right)\\ +℩\left(\log_{ϜℲ}\left(1+\prod_{\varepsilon =1}^{u}{\left({ϜℲ}^{{{}^{\circ}C}_{\kappa_{CB-\varepsilon }}^{℩P}}-1\right)}^{\frac{W_{vy-1}\left(1+T\left(\kappa_{CB-\varepsilon }\right)\right)}{\sum_{\varepsilon =1}^{.\ddot{u}}W_{vy-1}\left(1+T\left(\kappa_{CB-\varepsilon }\right)\right)}}\right)\right),\\ -1+\log_{ϜℲ}\left(1+{\prod_{\varepsilon =1}^{u}\left({ϜℲ}^{1+{{}^{\circ}C}_{\kappa_{CB-\varepsilon }}^{RN}}-1\right)}^{\frac{W_{vy-1}\left(1+T\left(\kappa_{CB-\varepsilon }\right)\right)}{\sum_{\varepsilon =1}^{.u}W_{vy-1}\left(1+T\left(\kappa_{CB-\varepsilon }\right)\right)}}\right)\\ +℩\left(-1+\log_{ϜℲ}\left(1+\prod_{\varepsilon =1}^{u}{\left({ϜℲ}^{1+{{}^{\circ}C}_{\kappa_{CB-\varepsilon }}^{℩N}}-1\right)}^{\frac{W_{vy-1}\left(1+T\left(\kappa_{CB-\varepsilon }\right)\right)}{\sum_{\varepsilon =1}^{.\ddot{u}}W_{vy-1}\left(1+T\left(\kappa_{CB-\varepsilon }\right)\right)}}\right)\right),\\ \log_{ϜℲ}\left(1+\prod_{\varepsilon =1}^{u}{\left({ϜℲ}^{\xi_{\kappa_{CB-\varepsilon }}^{RP}}-1\right)}^{\frac{W_{vy-1}\left(1+T\left(\kappa_{CB-\varepsilon }\right)\right)}{\sum_{\varepsilon =1}^{.\ddot{u}}W_{vy-1}\left(1+T\left(\kappa_{CB-\varepsilon }\right)\right)}}\right)\\ +℩\left(\log_{ϜℲ}\left(1+\prod_{\varepsilon =1}^{u}{\left({ϜℲ}^{\xi_{\kappa_{CB-\varepsilon }}^{℩P}}-1\right)}^{\frac{W_{vy-1}\left(1+T\left(\kappa_{CB-\varepsilon }\right)\right)}{\sum_{\varepsilon =1}^{.\ddot{u}}W_{vy-1}\left(1+T\left(\kappa_{CB-\varepsilon }\right)\right)}}\right)\right) \end{array}\right)$$

$${Cir-BCFFPWG}_{t}\left(\kappa_{CB-1},\kappa_{CB-2},\ldots ,\kappa_{CB-u\;}\right)=\left(\begin{array}{c} \log_{ϜℲ}\left(1+{\prod_{\varepsilon =1}^{u}\left({ϜℲ}^{{{}^{\circ}C}_{\kappa_{CB-\varepsilon }}^{RP}}-1\right)}^{\frac{W_{vy-1}\left(1+T\left(\kappa_{CB-\varepsilon }\right)\right)}{\sum_{\varepsilon =1}^{.u}W_{vy-1}\left(1+T\left(\kappa_{CB-\varepsilon }\right)\right)}}\right)\\ +℩\left(\log_{ϜℲ}\left(1+{\prod_{\varepsilon =1}^{u}\left({ϜℲ}^{{{}^{\circ}C}_{\kappa_{CB-\varepsilon }}^{℩P}}-1\right)}^{\frac{W_{vy-1}\left(1+T\left(\kappa_{CB-\varepsilon }\right)\right)}{\sum_{\varepsilon =1}^{.u}W_{vy-1}\left(1+T\left(\kappa_{CB-\varepsilon }\right)\right)}}\right)\right),\\ -1+\log_{ϜℲ}\left(1+\prod_{\varepsilon =1}^{u}{\left({ϜℲ}^{1+{{}^{\circ}C}_{\kappa_{CB-\varepsilon }}^{RN}}-1\right)}^{\frac{W_{vy-1}\left(1+T\left(\kappa_{CB-\varepsilon }\right)\right)}{\sum_{\varepsilon =1}^{.\ddot{u}}W_{vy-1}\left(1+T\left(\kappa_{CB-\varepsilon }\right)\right)}}\right)\\ +℩\left(-1+\log_{ϜℲ}\left(1+{\prod_{\varepsilon =1}^{u}\left({ϜℲ}^{1+{{}^{\circ}C}_{\kappa_{CB-\varepsilon }}^{RN}}-1\right)}^{\frac{W_{vy-1}\left(1+T\left(\kappa_{CB-\varepsilon }\right)\right)}{\sum_{\varepsilon =1}^{.\ddot{u}}W_{vy-1}\left(1+T\left(\kappa_{CB-\varepsilon }\right)\right)}}\right)\right),\\ 1-\log_{ϜℲ}\left(1+\prod_{\varepsilon =1}^{u}{\left({ϜℲ}^{1-\xi_{\kappa_{CB-\varepsilon }}^{RP}}-1\right)}^{\frac{W_{vy-1}\left(1+T\left(\kappa_{CB-\varepsilon }\right)\right)}{\sum_{\varepsilon =1}^{.u}W_{vy-1}\left(1+T\left(\kappa_{CB-\varepsilon }\right)\right)}}\right)\\ +℩\left(1-\log_{ϜℲ}\left(1+{\prod_{\varepsilon =1}^{u}\left({ϜℲ}^{1-\xi_{\kappa_{CB-\varepsilon }}^{℩P}}-1\right)}^{\frac{W_{vy-1}\left(1+T\left(\kappa_{CB-\varepsilon }\right)\right)}{\sum_{\varepsilon =1}^{.u}W_{vy-1}\left(1+T\left(\kappa_{CB-\varepsilon }\right)\right)}}\right)\right) \end{array}\right)$$

Proof *Omitted*.

*Consider two sets of Cir-BCFNs i*.*e*. $\kappa_{CB-\varepsilon }=\left({{}^{\circ}C}_{\kappa_{CB-\varepsilon }}^{P},{{}^{\circ}C}_{\kappa_{CB-\varepsilon }}^{N},\xi_{\kappa_{CB-\varepsilon }}^{P}\right)=\left({{}^{\circ}C}_{\kappa_{CB-\varepsilon }}^{RP}+℩{{}^{\circ}C}_{\kappa_{CB-\varepsilon }}^{℩P},{{}^{\circ}C}_{\kappa_{CB-\varepsilon }}^{RN}+℩{{}^{\circ}C}_{\kappa_{CB-\varepsilon }}^{℩N},\xi_{\kappa_{CB-\varepsilon }}^{RP}+℩\xi_{\kappa_{CB-\varepsilon }}^{℩P}\right)$*and*$\kappa_{CB-\varepsilon }^{/}=\left({{}^{\circ}C}_{\kappa_{CB-\varepsilon }}^{P},{{}^{\circ}C}_{\kappa_{CB-\varepsilon }}^{N},\xi_{\kappa_{CB-\varepsilon }}^{P}\right)=\left({{}^{\circ}C}_{\kappa_{CB-\varepsilon }}^{RP}+℩{{}^{\circ}C}_{\kappa_{CB-\varepsilon }}^{℩P},{{}^{\circ}C}_{\kappa_{CB-\varepsilon }}^{RN}+℩{{}^{\circ}C}_{\kappa_{CB-\varepsilon }}^{℩N},\xi_{\kappa_{CB-\varepsilon }}^{RP}+℩\xi_{\kappa_{CB-\varepsilon }}^{℩P}\right)$*then Cir-BFFPWG operator has underneath properties*.

Property 10 *Idempotency*: ∀ε*if*$\kappa_{CB-\varepsilon }=\kappa_{CB}$, *then*

$${Cir-BCFFPWG}_{tc}\left(\kappa_{CB-1},\kappa_{CB-2},\ldots ,\kappa_{CB-\ddot{u}}\right)=\kappa_{CB}$$

$${Cir-BCFFPWG}_{t}\left(\kappa_{CB-1},\kappa_{CB-2},\ldots ,\kappa_{CB-\ddot{u}}\right)=\kappa_{CB}$$

Property 11 *Boundedness*: *Assume that*$\kappa_{CB}^{-}=\left(\min_{\varepsilon }\left({{}^{\circ}C}_{\kappa_{CB-\varepsilon }}^{RP}\right)+℩\min_{\varepsilon }\left({{}^{\circ}C}_{\kappa_{CB-\varepsilon }}^{℩P}\right),\max_{\varepsilon }\left({{}^{\circ}C}_{\kappa_{CB-\varepsilon }}^{RN}\right)+℩\max_{\varepsilon }\left({{}^{\circ}C}_{\kappa_{CB-\varepsilon }}^{℩N}\right),\min_{\varepsilon }\left(\xi_{\kappa_{CB-\varepsilon }}^{RP}\right)+℩\min_{\varepsilon }\left(\xi_{\kappa_{CB-\varepsilon }}^{℩P}\right)\right)$*and*$\kappa_{CB}^{+}=\left(\max_{\varepsilon }\left({{}^{\circ}C}_{\kappa_{CB-\varepsilon }}^{RP}\right)+℩\max_{\varepsilon }\left({{}^{\circ}C}_{\kappa_{CB-\varepsilon }}^{℩P}\right),\min_{\varepsilon }\left({{}^{\circ}C}_{\kappa_{CB-\varepsilon }}^{RN}\right)+℩\min_{\varepsilon }\left({{}^{\circ}C}_{\kappa_{CB-\varepsilon }}^{℩N}\right),\max_{\varepsilon }\left(\xi_{\kappa_{CB-\varepsilon }}^{RP}\right)+℩\max_{\varepsilon }\left(\xi_{\kappa_{CB-\varepsilon }}^{℩P}\right)\right)$*for t-conorm and similarly for t-norm*, *then*

$$\kappa_{CB}^{-}\leq {Cir-BCFFPWG}_{tc}\left(\kappa_{CB-1},\kappa_{CB-2},\ldots ,\kappa_{CB-\ddot{u}}\right)\leq \kappa_{CB}^{+}$$

$$\kappa_{CB}^{-}\leq {Cir-BCFFPWG}_{t}\left(\kappa_{CB-1},\kappa_{CB-2},\ldots ,\kappa_{CB-\ddot{u}}\right)\leq \kappa_{CB}^{+}$$

Property 12 *Monotonicity*: ∀ε*if*$\kappa_{CB-\varepsilon }\leq K_{CB}^{/}$, *then*

$${Cir-BCFFPG}_{tc}\left(\kappa_{CB-1},\kappa_{CB-2},\ldots ,\kappa_{CB-\ddot{u}}\right)\leq {Cir-BCFFPWG}_{tc}\left(K_{CB-1}^{/},K_{CB-2}^{/},\ldots ,K_{CB-\ddot{u}}^{/}\right)$$

$${Cir-BCFFPG}_{t}\left(\kappa_{CB-1},\kappa_{CB-2},\ldots ,\kappa_{CB-\ddot{u}}\right)\leq {Cir-BCFFPWG}_{t}\left(K_{CB-1}^{/},K_{CB-2}^{/},\ldots ,K_{CB-\ddot{u}}^{/}\right)$$

## 5 EDAS method using proposed operator

The evaluation of the EDAS technique under the consideration of any kind of operators and measures is very complex, anyhow, in this section, we aim to compute the technique of the EDAS method based on proposed operators. Some dominant steps of the EDAS technique are listed below:

Step 1: We aim to construct the matrix by including their values in the shape of Cir-BCFSs.
Step 2: Find the aggregated values of the decision matrix by using the proposed operators, called the Cir-BCFFPA operator, such as

$${Cir-BCFFPA}_{tc}\left(\kappa_{CB-1},\kappa_{CB-2},\ldots ,\kappa_{CB-u\;}\right)=\left(\begin{array}{c} 1-\log_{ϜℲ}\left(1+\prod_{\varepsilon =1}^{u}{\left({ϜℲ}^{1-{{}^{\circ}C}_{\kappa_{CB-\varepsilon }}^{RP}}-1\right)}^{\frac{\left(1+T\left(\kappa_{CB-\varepsilon }\right)\right)}{\sum_{\varepsilon =1}^{.u}\left(1+T\left(\kappa_{CB-\varepsilon }\right)\right)}}\right)\\ +℩\left(1-\log_{ϜℲ}\left(1+\prod_{\varepsilon =1}^{u}{\left({ϜℲ}^{1-{{}^{\circ}C}_{\kappa_{CB-\varepsilon }}^{℩P}}-1\right)}^{\frac{\left(1+T\left(\kappa_{CB-\varepsilon }\right)\right)}{\sum_{\varepsilon =1}^{.u}\left(1+T\left(\kappa_{CB-\varepsilon }\right)\right)}}\right)\right),\\ -\left(\log_{ϜℲ}\left(1+\prod_{\varepsilon =1}^{u}{\left({ϜℲ}^{-{{}^{\circ}C}_{\kappa_{CB-\varepsilon }}^{RN}}-1\right)}^{\frac{\left(1+T\left(\kappa_{CB-\varepsilon }\right)\right)}{\sum_{\varepsilon =1}^{.u}\left(1+T\left(\kappa_{CB-\varepsilon }\right)\right)}}\right)\right)\\ +℩\left(-\left(\log_{ϜℲ}\left(1+\prod_{\varepsilon =1}^{u}{\left({ϜℲ}^{-{{}^{\circ}C}_{\kappa_{CB-\varepsilon }}^{℩N}}-1\right)}^{\frac{\left(1+T\left(\kappa_{CB-\varepsilon }\right)\right)}{\sum_{\varepsilon =1}^{.u}\left(1+T\left(\kappa_{CB-\varepsilon }\right)\right)}}\right)\right)\right),\\ 1-\log_{ϜℲ}\left(1+\prod_{\varepsilon =1}^{u}{\left({ϜℲ}^{1-\xi_{\kappa_{CB-\varepsilon }}^{RP}}-1\right)}^{\frac{\left(1+T\left(\kappa_{CB-\varepsilon }\right)\right)}{\sum_{\varepsilon =1}^{.u}\left(1+T\left(\kappa_{CB-\varepsilon }\right)\right)}}\right)\\ +℩\left(1-\log_{ϜℲ}\left(1+\prod_{\varepsilon =1}^{u}{\left({ϜℲ}^{1-\xi_{\kappa_{CB-\varepsilon }}^{℩P}}-1\right)}^{\frac{\left(1+T\left(\kappa_{CB-\varepsilon }\right)\right)}{\sum_{\varepsilon =1}^{.u}\left(1+T\left(\kappa_{CB-\varepsilon }\right)\right)}}\right)\right) \end{array}\right)$$

Step 3: Evaluate the PDA and NDA, according to the above information, such as

$${PDA}_{℩J}=\frac{\max\;(Ο,\left({l_{ij}-Cir-BCFFPA}_{\omega }^{tc}\left(Y_{j}\right)\right)}{{Cir-BCFFPA}_{\omega }^{tc}\left(Y_{j}\right)}$$

$${NDA}_{ij}=\frac{\max\;\left(Ο,\left({l_{ij}-Cir-BCFFPA}_{\omega }^{tc}\left(Y_{j}\right)\right)\right)}{{Cir-BCFFPA}_{\omega }^{tc}\left(Y_{j}\right)}$$

where both techniques will be used for all grades.

Step 4: Find the weighted summation using the obtained values from PDA and NDA, such as

$$Sum\left(P_{i}\right)=\frac{1}{n}\sum_{j=1}^{m}G_{j}\;\left({PDA}_{ij}\right)$$

$$Sum\left(N_{i}\right)=\frac{1}{n}\sum_{j=1}^{m}G_{j}\;\left({NDA}_{ij}\right)$$

Step 5: Find the normalized value with the help of the $Sum\left(P_{i}\right)$ and $Sum\left(N_{i}\right)$, such as

$$N\left(Sum\left(P_{i}\right)\right)=\frac{Sum\left(P_{i}\right)}{\max\left(Sum\left(P_{i}\right)\right)}$$

$$N\left(Sum\left(N_{i}\right)\right)=\frac{Sum\left(N_{i}\right)}{\max\left(Sum\left(N_{i}\right)\right)}$$

**Step 6:** Find the appraisal score, such as

$$ASCO\left({i_{῟}}_{i}\right)=\frac{1}{C}\left(N\left(Sum\left(P_{i}\right)\right)+N\left(Sum\left(N_{i}\right)\right)\right)$$

**Step 7**: Find the score value, such as

$$SC\left(T_{i}\right)=\frac{1}{6}\left(\psi_{\xi }^{+}+\mu_{\xi }^{+}+\psi_{\xi }^{-}+\mu_{\xi }^{-}+\psi_{\xi }^{+}+r_{\xi }^{+}\right)\in \left[-1,1\right]$$

if the score function has failed (if we obtained the same score values for all alternatives), then we will use the accuracy function, such as

$$H_{B}\left(\kappa_{CB}\right)=\frac{1}{6}\left({{}^{\circ}C}_{\kappa_{CB}}^{RP}+{{}^{\circ}C}_{\kappa_{CB}}^{℩P}-{{}^{\circ}C}_{\kappa_{CB}}^{RN}-{{}^{\circ}C}_{\kappa_{CB}}^{℩N}+\xi_{\kappa_{CB}}^{RP}+\xi_{\kappa_{CB}}^{℩P}\right),H_{B}\left(\kappa_{CB}\right)\in \left[O,1\right]$$

**Step 8:** Find the ranking results to examine the best one.

## 6 Application: analysis of renewable energy resources

In this section, we simplify the application of renewable energy resources [1,2] or analyze the analysis of renewable energy resources with the help of presented operators, where renewable energy resources are sources or techniques of energy that are refilled naturally and can be employed without reducing their reserves. Further, using the technique of MADM problems, we aim to evaluate the problem of renewable energy resources based on initiated operators. For this, we aim to consider some alternatives which represent different kinds of sources of renewable energy, such as

1) **Wind Energy (**$\kappa_{CB-1}$**):** Wind energy is a well-known part of renewable energy that harnesses the power of wind to generate electricity, some valuable components of wind energy are as follows, for instance, wind turbines, rotor blades, generators, towers, and control systems.
2) **Solar Energy (**$\kappa_{CB-2}$**):** Solar energy is famous for that energy can derived from the sun's radiations, some valuable components of solar energy are as follows, for instance, photovoltaics, Solar thermal systems, solar panels, inverters, net metering, and solar energy potential.
3) **Hydropower (**$\kappa_{CB-3}$**):** Hydropower is also used as a hydroelectric power and is famous for harnessing the energy of flowing or falling water to generate energy, some valuable components of hydropower is follows, for instance, dams and reservoirs, penstocks, turbines, and generators. Further, we have two kinds of hydropower systems, called run-of-river and reservoir storage.
4) **Geothermal Energy (**$\kappa_{CB-4}$**):** Geothermal energy is famous for deriving the energy from heat stored beneath the earth's surface, some valuable components of geothermal energy are as follows, for instance, geothermal heat and geothermal reservoirs.
5) **Biomass Energy (**$\kappa_{CB-5}$**):** Biomass energy is a famous renewable energy used for deriving energy from organic materials, some valuable components of biomass energy are as follows, for instance, feedstocks and conversion processes.

Further, we will use the following information as an attribute, such as growth analysis, social impact, political impact, environmental impact, and population ratio. Then, we will solve the above problem with the help of the EDAS method. Some dominant steps of the EDAS technique are listed below:

Step 1: We aim to construct the matrix by including their values in the shape of Cir-BCFSs, see Table 2.

**Table 2:** Cir-BCF decision matrix.

	$\kappa_{AT-1}$	$\kappa_{AT-2}$	$\kappa_{AT-3}$	$\kappa_{AT-4}$	$\kappa_{AT-5}$
$\kappa_{CB-1}$	$\left(\begin{array}{c} O.9+℩O.3\text{,}\\ -O.3-℩O.7\text{,}\\ O.3+℩O.3 \end{array}\right)$	$\left(\begin{array}{c} O.91+℩O.31\text{,}\\ -O.31-℩O.71\text{,}\\ O.31+℩O.31 \end{array}\right)$	$\left(\begin{array}{c} O.92+℩O.32\text{,}\\ -O.32-℩O.72\text{,}\\ O.32+℩O.32 \end{array}\right)$	$\left(\begin{array}{c} O.93+℩O.33\text{,}\\ -O.33-℩O.73\text{,}\\ O.33+℩O.33 \end{array}\right)$	$\left(\begin{array}{c} O.94+℩O.34\text{,}\\ -O.34-℩O.24\text{,}\\ O.34+℩O.34 \end{array}\right)$
$\kappa_{CB-2}$	$\left(\begin{array}{c} O.3+℩O.2\\ -O.3-℩O.8\\ O.4+℩O.4 \end{array}\right)$	$\left(\begin{array}{c} O.31+℩O.21\text{,}\\ -O.31-℩O.81\text{,}\\ O.41+℩O.41 \end{array}\right)$	$\left(\begin{array}{c} O.32+℩O.22\text{,}\\ -O.32-℩O.82\text{,}\\ O.42+℩O.42 \end{array}\right)$	$\left(\begin{array}{c} O.33+iO.23\text{,}\\ -O.33-℩O.83\text{,}\\ O.43+℩O.43 \end{array}\right)$	$\left(\begin{array}{c} O.34+℩O.24\text{,}\\ -O.34-℩O.24\text{,}\\ O.34+℩O.34 \end{array}\right)$
$\kappa_{CB-3}$	$\left(\begin{array}{c} O.5+℩O.2\text{,}\\ -O.6-℩O.7\\ O.3+℩O.3 \end{array}\right)$	$\left(\begin{array}{c} O.51+℩O.21\text{,}\\ -O.61,-℩O.71\text{,}\\ O.31+℩O.31 \end{array}\right)$	$\left(\begin{array}{c} O.52+℩O.22\text{,}\\ -O.62-℩O.72\text{,}\\ O.32+℩O.32 \end{array}\right)$	$\left(\begin{array}{c} O.53+℩O.23\text{,}\\ -O.63-℩O.73\text{,}\\ O.33+℩O.33 \end{array}\right)$	$\left(\begin{array}{c} O.94+℩O.34\text{,}\\ -O.64-℩O.74\text{,}\\ O.34+℩O.34 \end{array}\right)$
$\kappa_{CB-4}$	$\left(\begin{array}{c} O.1+℩O.3\text{,}\\ -O.5-℩O.8\text{,}\\ O.2+℩O.5 \end{array}\right)$	$\left(\begin{array}{c} O.11+℩O.31\text{,}\\ -O.51-℩O.81\text{,}\\ O.21+℩O.51 \end{array}\right)$	$\left(\begin{array}{c} Ο.12+℩Ο.32\text{,}\\ -Ο.52-℩Ο.82\text{,}\\ Ο.22+℩Ο.52 \end{array}\right)$	$\left(\begin{array}{c} Ο.13+iΟ.33\text{,}\\ -Ο.53-℩Ο.83\text{,}\\ Ο.23+iΟ.53 \end{array}\right)$	$\left(\begin{array}{c} O.14+℩O.34\text{,}\\ -O.54-℩O.84\text{,}\\ O.24+℩O.54 \end{array}\right)$
$\kappa_{CB-5}$	$\left(\begin{array}{c} O.4+℩O.7\text{,}\\ -O.6-℩O.9\\ O.1+℩O.4 \end{array}\right)$	$\left(\begin{array}{c} O.41+℩O.71\text{,}\\ -O.61-℩O.91\text{,}\\ O.11+℩O.41 \end{array}\right)$	$\left(\begin{array}{c} O.42+℩O.72\text{,}\\ -O.62-℩O.92\text{,}\\ O.12+℩O.42 \end{array}\right)$	$\left(\begin{array}{c} O.43+℩O.73\text{,}\\ -O.63-℩O.93\text{,}\\ O.13+℩O.43 \end{array}\right)$	$\left(\begin{array}{c} O.44+℩O.74\text{,}\\ -O.64-℩O.24\text{,}\\ O.34+℩O.34 \end{array}\right)$
Step 2: Find the aggregated values of the decision matrix by using the proposed operators, called the Cir-BCFFPA operator, see Table 3.

**Table 3:** Cir-BCF aggregated values.

	$Cir-{BCFFPA}_{tc}\;Operator$
$\kappa_{CB-1}$	$\left(\begin{array}{c} O.922O77+℩O.32O898\text{,}\\ -O.32O49-℩O.563O6\text{,}\\ O.32O898+℩O.32O898 \end{array}\right)$
$\kappa_{CB-2}$	$\left(\begin{array}{c} O.321238+℩O.221219\text{,}\\ -O.32O82-℩O.61784\\ O.397382+℩O.397382 \end{array}\right)$
$\kappa_{CB-3}$	$\left(\begin{array}{c} O.695159+℩O.246O11\text{,}\\ -O.62O69-℩O.72O71\text{,}\\ O.32O945+℩O.32O945 \end{array}\right)$
$\kappa_{CB-4}$	$\left(\begin{array}{c} O.12OO84+℩O.32O117\text{,}\\ -O.51984-℩O.81991\text{,}\\ O.22OO98+℩O.52O178 \end{array}\right)$
$\kappa_{CB-5}$	$\left(\begin{array}{c} O.421564+℩O.72176\text{,}\\ -O.62128-℩O.66745\\ O.177766+℩O.396412 \end{array}\right)$
Step 3: Evaluate the PDA and NDA, according to the above information, see Table 4.

**Table 4:** Representation of the PDA and NDA information.

$PDA_{ij}$	$\kappa_{AT-1}$	$\kappa_{AT-2}$	$\kappa_{AT-3}$	$\kappa_{AT-4}$	$\kappa_{AT-5}$
$\kappa_{CB-1}$	$\left(\begin{array}{c} O+℩O\text{,}\\ -O\\ -℩O.24321\text{,}\\ O+℩O \end{array}\right)$	$\left(\begin{array}{c} O+℩O\text{,}\\ -O\\ -℩O.26O97\text{,}\\ O+℩O \end{array}\right)$	$\left(-\begin{array}{c} O+℩O,\\ O-℩O.27873,\\ O+℩O \end{array}\right)$	$\left(\begin{array}{c} O.OO8593\\ +℩O.O28354\text{,}\\ O.O2969\\ +℩O.27873\text{,}\\ O.O28364\\ +℩O‥O28354 \end{array}\right)$	$\left(\begin{array}{c} O.O19438\\ +℩O.O59526\text{,}\\ -O.O6O89\\ -℩O\text{,}\\ O.O59526\\ +℩O.O59526 \end{array}\right)$
$\kappa_{CB-2}$	$\left(\begin{array}{c} O+℩O\text{,}\\ -O\\ -℩O.29484\text{,}\\ O.OO6587\\ +℩O.OO6587 \end{array}\right)$	$\left(\begin{array}{c} O+℩O\text{,}\\ -O\\ -℩O.311O3\text{,}\\ O.O31752\\ +℩O.O31752 \end{array}\right)$	$\left(\begin{array}{c} O+℩O\text{,}\\ -O\\ -℩O.32721\text{,}\\ O.O56916\\ +℩O.O56916 \end{array}\right)$	$\left(\begin{array}{c} O.O27275\\ +℩O.O39694\text{,}\\ -O.O286\\ -℩O.32721\text{,}\\ O.O82O81\\ +℩O.O82O81 \end{array}\right)$	$\left(\begin{array}{c} O.O584O4\\ +℩O.O84898\text{,}\\ -O.O5977\\ +℩O\text{,}\\ O\\ +℩O \end{array}\right)$
$\kappa_{CB-3}$	$\left(\begin{array}{c} O\\ +℩O\text{,}\\ -O\\ -℩O\text{.}\\ O+℩O \end{array}\right)$	$\left(\begin{array}{c} O\\ +℩O\text{,}\\ -O\\ -iO\text{,}\\ O\\ +℩O \end{array}\right)$	$\left(\begin{array}{c} O\\ +℩O\text{,}\\ -O\\ -℩O\text{,}\\ O\\ +℩O \end{array}\right)$	$\left(\begin{array}{c} O\\ +℩O\text{,}\\ -O.O15\\ -℩O\text{,}\\ Ο.O28213\\ +℩O.O28213 \end{array}\right)$	$\left(\begin{array}{c} O.3522O9\\ +℩O.382O52\text{,}\\ -O.O3111\\ -℩O.O2676.,\\ O.O59371\text{.}\\ +℩O.O59371 \end{array}\right)$
$\kappa_{CB-4}$	$\left(\begin{array}{c} O\\ +℩O\text{,}\\ -O\\ -℩O\text{,}\\ O\\ +℩O \end{array}\right)$	$\left(\begin{array}{c} O\\ +℩O\text{,}\\ -O\\ -℩O\text{,}\\ O\\ +℩O \end{array}\right)$	$\left(\begin{array}{c} O\\ +℩O\text{,}\\ -O.OOO31\\ -℩O.OOO11\text{,}\\ O\\ +℩O \end{array}\right)$	$\left(\begin{array}{c} O.O82579\\ +℩O.O3O874\text{,}\\ -O.O1955\\ -℩O.OOO11\text{,}\\ ℩.O44989\\ +℩O.O18882 \end{array}\right)$	$\left(\begin{array}{c} O.165855\\ +℩O.O62113\text{,}\\ -O.O3878\\ -℩O.O245.,\\ O.O9O424\text{.}\\ +℩O.O381O6 \end{array}\right)$
$\kappa_{CB-5}$	$\left(\begin{array}{c} O\\ +℩O\text{,}\\ -O\\ -℩O.34842\\ O\\ +℩O.OO9O52 \end{array}\right)$	$\left(\begin{array}{c} O\\ +℩O\text{,}\\ -O\\ -℩O.36341\text{,}\\ O\\ +℩O.O34278 \end{array}\right)$	$\left(\begin{array}{c} O\\ +℩O\text{,}\\ -O\\ -℩O.37839\text{,}\\ O\\ +℩O.O595O5 \end{array}\right)$	$\left(\begin{array}{c} O.O2OO12\\ +℩O.Ο11416\text{,}\\ -O.O14O4\\ +℩O.37839\text{,}\\ Ο+℩O.O84731 \end{array}\right)$	$\left(\begin{array}{c} O.O43733\\ +℩Ο.O25271\text{,}\\ -Ο.O3O14\\ +℩Ο\text{,}\\ Ο.912628\\ +℩Ο \end{array}\right)$
$NDA_{ij}$	$\kappa_{AT-1}$	$\kappa_{AT-2}$	$\kappa_{AT-3}$	$\kappa_{AT-4}$	$\kappa_{AT-5}$
$\kappa_{CB-1}$	$\left(\begin{array}{c} O.O23943\\ +℩O.O65124\text{,}\\ -O.O6392\\ -℩O\text{,}\\ O.O65124\\ +℩O.O65124 \end{array}\right)$	$\left(\begin{array}{c} O.O13O97\\ +℩O.O33961\text{,}\\ -O.O3272\\ -℩O\text{,}\\ O.O33961\\ +℩O.O33961 \end{array}\right)$	$\left(\begin{array}{c} O.OO2252\\ +℩O.OO2799\text{,}\\ -O.OO152\\ -℩O\text{,}\\ O.OO2799\\ +℩O.OO2799 \end{array}\right)$	$\left(\begin{array}{c} O\\ +℩O\text{,}\\ -O\\ -℩O\text{,}\\ O\\ +℩O \end{array}\right)$	$\left(\begin{array}{c} O\\ +℩O\text{,}\\ -O.O48O6\\ -℩O.O2786\text{,}\\ O.24424\\ +℩O.O66697 \end{array}\right)$
$\kappa_{CB-2}$	$\left(\begin{array}{c} O.O66114\\ +℩O.O95918\text{,}\\ -O.O6491\\ -℩O\text{,}\\ O\\ +℩O \end{array}\right)$	$\left(\begin{array}{c} O.O34984\\ +℩O.O5O714\text{,}\\ -O.O3374\\ -℩O\text{,}\\ O\\ +℩O \end{array}\right)$	$\left(\begin{array}{c} O.OO3855\\ +℩O.OO551\text{,}\\ -O.OO257\\ -℩O\text{,}\\ O\\ +℩O \end{array}\right)$	$\left(\begin{array}{c} O\\ +℩O\text{,}\\ -O\\ -℩O\text{,}\\ O\\ +℩O \end{array}\right)$	$\left(\begin{array}{c} O\\ +℩O\text{,}\\ -O\\ -℩O.61155\text{,}\\ O.1444O1\\ +℩O.1444O1 \end{array}\right)$
$\kappa_{CB-3}$	$\left(\begin{array}{c} O.28O74\\ +℩O.187O28\text{,}\\ -O.O3333\\ -℩O.O2874\text{,}\\ O.O65261\\ +℩O.O65261 \end{array}\right)$	$\left(\begin{array}{c} O.266355\\ +℩O.14638\text{,}\\ -O.O1722\\ -℩O.O1486\text{,}\\ O.O341O3\\ +℩O.O341O3 \end{array}\right)$	$\left(\begin{array}{c} O.25197\\ +℩O.1O5731\text{,}\\ -O.OO111\\ -℩O.OOO99\text{,}\\ O.OO2945\\ +℩O.OO2945 \end{array}\right)$	$\left(\begin{array}{c} O.237585\\ +℩O.O65O83\text{,}\\ -O\\ -℩O\text{,}\\ O\\ +℩O \end{array}\right)$	$\left(\begin{array}{c} O\\ +℩O\text{,}\\ -O\\ -℩O\text{,}\\ O\\ +℩O \end{array}\right)$
$\kappa_{CB-4}$	$\left(\begin{array}{c} O.167247\\ +℩O.O62842\\ -O.O3816\\ -℩O.O2428\text{,}\\ O.O91314\\ +℩O.O38791 \end{array}\right)$	$\left(\begin{array}{c} O.O83971\\ +℩O.O316O3\text{,}\\ -O.O1893\\ -℩O.O12O8\text{,}\\ O.O45879\\ +℩O.O19566 \end{array}\right)$	$\left(\begin{array}{c} O.OOO696\\ +℩O.OOO364\\ -Ο\\ -℩O\text{,}\\ O.OOO445\\ +℩O.OOOO342 \end{array}\right)$	$\left(\begin{array}{c} O\\ +℩O\text{,}\\ -O\\ -℩O\text{,}\\ O\\ +℩O \end{array}\right)$	$\left(\begin{array}{c} O\\ +℩O\text{,}\\ -O\\ -℩O\text{,}\\ O\\ +℩O \end{array}\right)$
$\kappa_{CB-5}$	$\left(\begin{array}{c} O.O51152\\ +℩O.O3O149\text{,}\\ -O.O3425\\ -℩O\text{,}\\ O.437462\\ +℩O \end{array}\right)$	$\left(\begin{array}{c} O.O27431\\ +℩O.O16294\text{,}\\ O.O1815\\ +℩O\text{,}\\ O.3812O9\\ +℩O \end{array}\right)$	$\left(\begin{array}{c} O.OO37O9\\ +℩O.OO2439\text{,}\\ -O.OO2O5\\ -℩O\text{,}\\ O.324955\\ +℩O \end{array}\right)$	$\left(\begin{array}{c} O\\ +℩O\text{,}\\ -O\\ -℩O\text{,}\\ O.2687O1\\ +℩O \end{array}\right)$	$\left(\begin{array}{c} O\\ +℩O\text{,}\\ -O\\ -℩O.64O42\text{,}\\ O\\ +℩O.1423O6 \end{array}\right)$
Step 4: Find the weighted summation using the obtained values from PDA and NDA, see Table 5.

**Table 5:** Representation of the weighted summation values.

Alternatives	$Sum\left(P_{i}\right)$	$Sum\left(N_{i}\right)$
$\kappa_{CB-1}$	$\left(\begin{array}{c} O.OO1121\\ +℩O.OO3516\text{,}\\ -O.OO362-\\ iO.O4247\text{,}\\ O.OO3516\\ +℩O.OO3516 \end{array}\right)$	$\left(\begin{array}{c} Ο.ΟΟ1572\\ +iΟ.ΟO4O75\text{,}\\ -Ο.ΟO393-\\ iΟ.Ο2295\text{,}\\ Ο.OO4O75\\ +iΟ.OO4O75 \end{array}\right)$
$\kappa_{CB-2}$	$\left(\begin{array}{c} O.OO3427\\ +℩O.OO4984\text{,}\\ -O.OO354\\ -℩O.O5O41\text{,}\\ O.OO683\\ +℩O.OO7O93 \end{array}\right)$	$\left(\begin{array}{c} O.OO4198\\ +℩O.OO6O86\text{,}\\ -O.OO4O5-\\ ℩O.O2446\text{,}\\ O.OO5776\\ +℩O.OO5776 \end{array}\right)$
$\kappa_{CB-3}$	$\left(\begin{array}{c} O.O14O88\\ +℩O.O15282\text{,}\\ -O.OO184-\\ ℩O.OO1O7\text{,}\\ O.OO35O3\\ +℩O.OO35O3 \end{array}\right)$	$\left(\begin{array}{c} O.O414668\\ +℩O.O2O169\text{,}\\ -O.OO2O7-\\ ℩O.OO178\text{,}\\ O.OO4O92\\ +℩O.OO4O92 \end{array}\right)$
$\kappa_{CB-4}$	$\left(\begin{array}{c} O.OO9937\\ +℩O.OO3719\text{,}\\ -O.OO235-\\ ℩O.OOO99\text{,}\\ O.OO54171\\ +℩O.OO228 \end{array}\right)$	$\left(\begin{array}{c} O.O1OO77\\ +℩O.OO3792\text{,}\\ -O.OO228\\ -℩O.OO145\\ O.OO55O6\\ +℩O.22939 \end{array}\right)$
Step 5: Find the normalized value with the help of the $Sum\left(P_{i}\right)$ and $Sum\left(N_{i}\right)$, see Table 6.

**Table 6:** Representation of the normalized matrix.

Alternatives	$N\left(Sum\left(P_{i}\right)\right)$	$N\left(Sum\left(N_{i}\right)\right)$
$\kappa_{CB-1}$	$\left(\begin{array}{c} O.318928+℩1\text{,}\\ -O.OO361-℩O.O4232\text{,}\\ 1+℩1 \end{array}\right)$	$\left(\begin{array}{c} Ο.385657+℩1\text{,}\\ -Ο.OO394-℩O.O23O4\text{,}\\ 1+℩1 \end{array}\right)$
$\kappa_{CB-2}$	$\left(\begin{array}{c} -Ο.483145-℩Ο.7O2581\text{,}\\ -O.OO351-℩O.O5OO6\text{,}\\ -O.962856-℩1 \end{array}\right)$	$\left(\begin{array}{c} O.6892Ο7+℩1.,\\ -O.OO4O7-℩O.O2461\text{,}\\ O.949122+℩O.949122 \end{array}\right)$
$\kappa_{CB-3}$	$\left(\begin{array}{c} O.921887+℩1\text{,}\\ -O.OO182-℩O.OO1O5\text{,}\\ O.229248+℩O.229248 \end{array}\right)$	$\left(\begin{array}{c} 1+iO.486396\text{,}\\ -Ο.OO216-℩O.OO186\text{,}\\ Ο.O986911+℩Ο.O98691 \end{array}\right)$
$\kappa_{CB-4}$	$\left(\begin{array}{c} 1+℩Ο.O.374293\text{,}\\ -Ο.OO232-℩O.OOO98\text{,}\\ +O.545O66+℩O.22939 \end{array}\right)$	$\left(\begin{array}{c} 1+℩O.376355\\ -O.OO231-℩O.OO147\text{,}\\ O.546369+℩O.233O12 \end{array}\right)$
$\kappa_{CB-5}$	$\left(\begin{array}{c} O.O69848-iO.O4O199\text{,}\\ -O.OO17-℩O.O5668\text{,}\\ 1+℩O.2O5523 \end{array}\right)$	$\left(\begin{array}{c} O.O58267+℩O.Ο34611\text{,}\\ -O.OO231-℩O.O2715\text{,}\\ 1+℩O.1OO76 \end{array}\right)$
**Step 6:** Find the appraisal score, see Table 7.

**Table 7:** Representation of the appraisal score information.

Alternatives	Asco($\psi_{i}$)
$\kappa_{CB-1}$	$\left(\begin{array}{c} O.352292+℩1\text{,}\\ -O.OO378-℩O.O3268\text{,}\\ 1+℩1 \end{array}\right)$
$\kappa_{CB-2}$	$\left(\begin{array}{c} O.586492+℩O.851291\text{,}\\ -O.OO379-℩O.O3733\text{,}\\ O.955989-iO.974561 \end{array}\right)$
$\kappa_{CB-3}$	$\left(\begin{array}{c} O.96O944+℩O.743198\text{,}\\ -O.OO199-℩O.OO146\text{,}\\ -O.163969-℩O.163969\text{.} \end{array}\right)$
$\kappa_{CB-4}$	$\left(\begin{array}{c} 1+℩O.375324\text{,}\\ -O.OO231-℩O.OO122\text{,}\\ -O.545717-℩O.2312O1 \end{array}\right)$
$\kappa_{CB-5}$	$\left(\begin{array}{c} O.O64O57+℩O.O374O5\text{,}\\ -O.OO2O1.-℩Ο.O4191\text{,}\\ 1+℩O.153141 \end{array}\right)$
**Step 7**: Find the score value, see Table 8.

**Table 8:** Representation of the score information.

Alternatives	Score values
$\kappa_{CB-1}$	O.552639
$\kappa_{CB-2}$	0.554535
$\kappa_{CB-3}$	O.3381O6
$\kappa_{CB-4}$	O.358117
$\kappa_{CB-5}$	O.2O1781
**Step 8:** Find the ranking results to examine the best one, see Table 9.

**Table 9:** Representation of the ranking values.

Method	Ranking values	Good Optimal
EDAS Method	$\kappa_{CB-2}>\kappa_{CB-1}>\kappa_{CB-4}>\kappa_{CB-3}>\kappa_{CB-5}$	$\kappa_{CB-2}$

The most preferable technique is $\kappa_{CB-2}$, represented the Solar Energy. Further, we consider the information in Table 10, then we evaluate it with the help of initiated operators.

**Table 10:** 0Cir-BCF decision matrix.

	$\kappa_{AT-1}$	$\kappa_{AT-2}$	$\kappa_{AT-3}$	$\kappa_{AT-4}$	$\kappa_{AT-5}$
$\kappa_{CB-1}$	$\left(\begin{array}{c} O.9+℩O.3\text{,}\\ -O.3-℩O.7\text{,}\\ O.3+℩O.3 \end{array}\right)$	$\left(\begin{array}{c} O.91+℩O.31\text{,}\\ -O.31-℩O.71\text{,}\\ O.31+℩O.31 \end{array}\right)$	$\left(\begin{array}{c} O.92+℩O.32\text{,}\\ -O.32-℩O.72\text{,}\\ O.32+℩O.32 \end{array}\right)$	$\left(\begin{array}{c} O.93+℩O.33\text{,}\\ -O.33-℩O.73\text{,}\\ O.33+℩O.33 \end{array}\right)$	$\left(\begin{array}{c} O.94+℩O.34\text{,}\\ -O.34-℩O.24\text{,}\\ O.34+℩O.34 \end{array}\right)$
$\kappa_{CB-2}$	$\left(\begin{array}{c} O.3+℩O.2\\ -O.3-℩O.8\\ O.4+℩O.4 \end{array}\right)$	$\left(\begin{array}{c} O.31+℩O.21\text{,}\\ -O.31-℩O.81\text{,}\\ O.41+℩O.41 \end{array}\right)$	$\left(\begin{array}{c} O.32+℩O.22\text{,}\\ -O.32-℩O.82\text{,}\\ O.42+℩O.42 \end{array}\right)$	$\left(\begin{array}{c} O.33+iO.23\text{,}\\ -O.33-℩O.83\text{,}\\ O.43+℩O.43 \end{array}\right)$	$\left(\begin{array}{c} O.34+℩O.24\text{,}\\ -O.34-℩O.24\text{,}\\ O.34+℩O.34 \end{array}\right)$
$\kappa_{CB-3}$	$\left(\begin{array}{c} O.5+℩O.2\text{,}\\ -O.6-℩O.7\\ O.3+℩O.3 \end{array}\right)$	$\left(\begin{array}{c} O.51+℩O.21\text{,}\\ -O.61,-℩O.71\text{,}\\ O.31+℩O.31 \end{array}\right)$	$\left(\begin{array}{c} O.52+℩O.22\text{,}\\ -O.62-℩O.72\text{,}\\ O.32+℩O.32 \end{array}\right)$	$\left(\begin{array}{c} O.53+℩O.23\text{,}\\ -O.63-℩O.73\text{,}\\ O.33+℩O.33 \end{array}\right)$	$\left(\begin{array}{c} O.94+℩O.34\text{,}\\ -O.64-℩O.74\text{,}\\ O.34+℩O.34 \end{array}\right)$
$\kappa_{CB-4}$	$\left(\begin{array}{c} O.1+℩O.3\text{,}\\ -O.5-℩O.8\text{,}\\ O.2+℩O.5 \end{array}\right)$	$\left(\begin{array}{c} O.11+℩O.31\text{,}\\ -O.51-℩O.81\text{,}\\ O.21+℩O.51 \end{array}\right)$	$\left(\begin{array}{c} Ο.12+℩Ο.32\text{,}\\ -Ο.52-℩Ο.82\text{,}\\ Ο.22+℩Ο.52 \end{array}\right)$	$\left(\begin{array}{c} Ο.13+iΟ.33\text{,}\\ -Ο.53-℩Ο.83\text{,}\\ Ο.23+iΟ.53 \end{array}\right)$	$\left(\begin{array}{c} O.14+℩O.34\text{,}\\ -O.54-℩O.84\text{,}\\ O.24+℩O.54 \end{array}\right)$
$\kappa_{CB-5}$	$\left(\begin{array}{c} O.4+℩O.7\text{,}\\ -O.6-℩O.9\\ O.1+℩O.4 \end{array}\right)$	$\left(\begin{array}{c} O.41+℩O.71\text{,}\\ -O.61-℩O.91\text{,}\\ O.11+℩O.41 \end{array}\right)$	$\left(\begin{array}{c} O.42+℩O.72\text{,}\\ -O.62-℩O.92\text{,}\\ O.12+℩O.42 \end{array}\right)$	$\left(\begin{array}{c} O.43+℩O.73\text{,}\\ -O.63-℩O.93\text{,}\\ O.13+℩O.43 \end{array}\right)$	$\left(\begin{array}{c} O.44+℩O.74\text{,}\\ -O.64-℩O.24\text{,}\\ O.34+℩O.34 \end{array}\right)$

Further, by using the initiated operators, we have the following aggregated values, see Table 11.

**Table 11:** Cir-BCF aggregated matrix.

Operators	$\kappa_{CB-1}$	$\kappa_{CB-2}$	$\kappa_{CB-3}$
$C{ir-BCFFPA}_{tc}$	$\left(\begin{array}{c} O.922O77+℩O.32O898\text{,}\\ -O.32O49-℩O.563O6\text{,}\\ O.32O898+℩O.32O898 \end{array}\right)$	$\left(\begin{array}{c} O.321238+℩O.221219\text{,}\\ -O.32O82-℩O.61784\\ O.397382+℩O.397382 \end{array}\right)$	$\left(\begin{array}{c} O.695159+℩O.246O11\text{,}\\ -O.62O69-℩O.72O71\text{,}\\ O.32O945+℩O.32O945 \end{array}\right)$
${Cir-BCFFPA}_{t}$	$\left(\begin{array}{c} O.922O77+℩O.32O898\text{,}\\ -O.32O49-℩O.563O6\text{,}\\ O.32O49+℩O.563O6 \end{array}\right)$	$\left(\begin{array}{c} Ο.321238+℩O.221219\text{,}\\ -Ο.32O82-℩O.61784\text{,}\\ Ο.32O82+℩O.61784 \end{array}\right)$	$\left(\begin{array}{c} O.695159+℩O.246O11\text{,}\\ -O.62O69-℩O.72O71\text{,}\\ O.62O69+℩O.72O71 \end{array}\right)$
$Cir-{BCFFPG}_{tc}$	$\left(\begin{array}{c} O.O77923+℩O.6791O2\text{,}\\ -O.67951-℩O.43694\text{,}\\ O.32O898+℩O.32O898 \end{array}\right)$	$\left(\begin{array}{c} O.678762+℩O.778781\text{,}\\ -O.67918-℩O.38216\text{,}\\ O.397382+℩O.397382 \end{array}\right)$	$\left(\begin{array}{c} O.3O4841+℩O.753989\text{,}\\ -O.37931-℩O.27929\text{,}\\ O.32O945+℩O.32O945 \end{array}\right)$
$C{ir-BCFFPG}_{t}$	$\left(\begin{array}{c} O.O77923+℩O.6791O2\text{,}\\ -O.67951-℩O.43694\text{,}\\ O.6791O2+℩O.6791O2 \end{array}\right)$	$\left(\begin{array}{c} O.678762+℩O.778781\text{,}\\ -O.67918-℩O.38216\text{,}\\ O.6O2618+℩O.6O2618 \end{array}\right)$	$\left(\begin{array}{c} O.3O4841+℩O.753989\text{,}\\ -O.37931-℩O.27929\text{,}\\ O.679O55+℩O.679O55 \end{array}\right)$
Operators	$\kappa_{CB-4}$	$\kappa_{CB-5}$	
$C{Bir-CFFPA}_{tc}$	$\left(\begin{array}{c} O.12OO84+℩O.32O117,\\ -O.51984-℩O.81991,\\ O.22OO98+℩O.52O178 \end{array}\right)\left(\begin{array}{c} Ο.12OO84+iΟ.32O117,\\ -Ο.51984-iΟ.81991,\\ Ο.22OO98+iΟ.52O178 \end{array}\right)$	$\left(\begin{array}{c} O.421564+℩O.72176,\\ -O.62128-℩O.66745\\ O.177766+℩O.396412 \end{array}\right)\left(\begin{array}{c} Ο.421564+iΟ.72176,\\ -Ο.62128-iΟ.66745\\ Ο.177766+iΟ.396412 \end{array}\right)$	
${Cir-BCFFPA}_{t}$	$\left(\begin{array}{c} O.12OO84+℩O.32O117\text{,}\\ -O.51984-℩O.81991\text{,}\\ O.22OO98+℩O.52O178 \end{array}\right)$	$\left(\begin{array}{c} O.421564+℩O.72176,\\ -O.62128-℩O.66745\\ O.62128+℩O.66745 \end{array}\text{,}\right)$	
$Cir-{BCFFPG}_{tc}$	$\left(\begin{array}{c} O.879916+℩O.679883\text{,}\\ -O.48O16-℩O.18OO9\text{,}\\ O.22OO98+℩O.52O178 \end{array}\right)$	$\left(\begin{array}{c} O.578436+℩O.27824\text{,}\\ -O.37872-℩O.33255\text{,}\\ O.177766+℩O.396412 \end{array}\right)$	
${Cir-BCFFPG}_{t}$	$\left(\begin{array}{c} O.879916+℩O.679883,\\ -O.48O16-℩O.18OO9,\\ O.7799O2+℩O.479822 \end{array}\right)\left(\begin{array}{c} Ο.879916+iΟ.679883,\\ -Ο.48O16-iΟ.18OO9,\\ Ο.7799O2+iΟ.479822 \end{array}\right)$	$\left(\begin{array}{c} O.578436+℩O.27824\text{,}\\ -O.37872-℩O.33255\text{,}\\ O.822234+℩O.6O3588 \end{array}\right)$	

Using the score values, see Table 12.

**Table 12:** Cir-BCF score information.

Operators	$\kappa_{CB-1}$	$\kappa_{CB-2}$	$\kappa_{CB-3}$
$Cir-{BCFFPA}_{tc}$	O.16871	O.O66427	O.O4O277
${Cir-BCFFPA}_{t}$	O.2O7163	O.O9O41	O.156862
$Cir-{BCFFPG}_{tc}$	O.166462	O.2669O6	O.293O57
${Cir-BCFFPG}_{t}$	O.O47O61	O.198495	O.173687
Operators	$\kappa_{CB-4}$	$\kappa_{CB-5}$	
$C{ir-BCFFPA}_{tc}$	−O.O2655	O.O71463	
${Cir-BCFFPA}_{t}$	O.O73367	O.19O554	
$C{ir-BCFFPG}_{tc}$	O.359878	O.26187	
${Cir-BCFFPG}_{t}$	O.2733O4	O.119929	

Using the data in Table 12, the ranking values are stated in Table 13.

**Table 13:** Representation of the ranking values.

Operators	Ranking
$C{ir-BCFFPA}_{tc}$	$\kappa_{CB-1}>\kappa_{CB-5}>\kappa_{CB-2}>\kappa_{CB-3}>\kappa_{CB-4}$
${Cir-BCFFPA}_{t}$	$\kappa_{CB-1}>\kappa_{CB-5}>\kappa_{CB-3}>\kappa_{CB-2}>\kappa_{CB-4}$
$Cir-{BCFFPG}_{tc}$	$\kappa_{CB-4}>\kappa_{CB-3}>\kappa_{CB-2}>\kappa_{CB-5}>\kappa_{CB-1}$
${Cir-BCFFPG}_{t}$	$\kappa_{CB-4}>\kappa_{CB-3}>\kappa_{CB-2}>\kappa_{CB-5}>\kappa_{CB-1}$

The most preferable technique is $\kappa_{CB-1}$, according to the theory of Cir-BCFFPA for t-norm and t-conorm, and similarly, the best decision is $\kappa_{CB-4}$, according to the theory of Cir-BCFFPG for t-norm and t-conorm. Further, we find the comparison between proposed and existing ranking values to show the supremacy and validity of the proposed theory.

## 7 Comparative analysis

This section contained the comparison between proposed operators and existing techniques by evaluating the information in Table 1. The evaluation of the comparison in every manuscript is very effective and reliable because of their features. With the help of comparative analysis, we can easily determine the supremacy and validity of the initiated operators. For comparison, we will consider some prevailing techniques, such as aggregation operators based on FSs initiated by Yager [24]. Further, Bi et al. [25] exposed the technique of arithmetic operators for CFSs. In 2018, the technique of geometric operators for CFSs was proposed by Bi et al. [26]. Additionally, Hu et al. [27] presented the power operators for CFSs. Moreover, Mahmood et al. [28] identified the classification of the aggregation operators for BCFSs and their applications. In 2021, The technique of Hamacher operators for BCFSs was initiated by Mahmood et al. [29]. Further, frank operators for BCFSs were initiated by Naeem et al. [30]. The model of Aczel-Alsina information based on the Cir-BCF set (Cir-BCFS) was presented by Ali et al. [31]. Using the data in Table 2, the comparative analysis is listed in Table 14.

**Table 14:** Representation of the comparative analysis.

Methods	Ranking values	Best optimal
Yager [24]	Failed	No
Bi et al. [25]	Failed	No
Bi et al. [26]	Failed	No
Hu et al. [27]	Failed	No
Mahmood et al. [28]	Failed	No
Mahmood et al. [29]	Failed	No
Naeem et al. [30]	Failed	No
Ali et al. [31]	$\kappa_{CB-1}>\kappa_{CB-5}>\kappa_{CB-2}>\kappa_{CB-3}>\kappa_{CB-4}$	$\kappa_{CB-1}$
$C{ir-BCFFPA}_{tc}$	$\kappa_{CB-1}>\kappa_{CB-5}>\kappa_{CB-2}>\kappa_{CB-3}>\kappa_{CB-4}$	$\kappa_{CB-1}$
${Cir-BCFFPA}_{t}$	$\kappa_{CB-1}>\kappa_{CB-5}>\kappa_{CB-3}>\kappa_{CB-2}>\kappa_{CB-4}$	$\kappa_{CB-1}$
$Cir-{BCFFPG}_{tc}$	$\kappa_{CB-4}>\kappa_{CB-3}>\kappa_{CB-2}>\kappa_{CB-5}>\kappa_{CB-1}$	$\kappa_{CB-4}$
${Cir-BCFFPG}_{t}$	$\kappa_{CB-4}>\kappa_{CB-3}>\kappa_{CB-2}>\kappa_{CB-5}>\kappa_{CB-1}$	$\kappa_{CB-4}$

The most preferable technique is $\kappa_{CB-1}$, according to the theory of Cir-BCFFPA for t-norm and t-conorm, and similarly, the best decision is $\kappa_{CB-4}$, according to the theory of Cir-BCFFPG for t-norm and t-conorm. Further, the proposed theory of Ali et al. [31] also gives the same ranking values as the Cir-BCFFPA operator. But, the prevailing techniques have failed because of various problems and limitations. Further, we discussed the limitations and problems of the existing techniques with the help of qualitative analysis in Table 15.

**Table 15:** Qualitative comparison between proposed and existing techniques.

Methods	Aggregation	Falsity (negative membership function)	Methods	Periodicity	Ability to model multi-dimensional data	Radius function
Yager [24]	√	×	×	×	×	×
Bi et al. [25]	√	×	×	√	×	×
Bi et al. [26]	√	×	×	√	×	×
Hu et al. [27]	√	×	×	√	×	×
Mahmood et al. [28]	√	√	×	√	×	×
Mahmood et al. [29]	√	√	×	√	×	×
Naeem et al. [30]	√	√	×	√	×	×
Ali et al. [31]	√	√	×	√	√	×
$C{ir-BCFFPA}_{tc}$	√	√	√	√	√	√
${Cir-BCFFPA}_{t}$	√	√	√	√	√	√
$Cir-{BCFFPG}_{tc}$	√	√	√	√	√	√
${Cir-BCFFPG}_{t}$	√	√	√	√	√	√

The term “√” is used for yes and the symbol “×” is used for no, from the information in Table 15, we observed that the proposed techniques are very famous and accurate in evaluating the information in Table 2, but the existing technique has covered a lot of problems.

Hence, the proposed theory is novel and up to date no one can propose it yet, anyhow, the initiated techniques are reliable and effective due to their features.

## 8 Conclusion

The model of Cir-BCF sets is computed based on the membership function, non-membership function, and radius among both functions for each value of the universal set. Based on the supremacy and validity of the Cir-BCFSs, frank information, EDAS model, and power operators, we concluded the following remarks, such as for developing any kind of operators, we described the novel technique of frank operational laws based on Cir-BCF values for frank t-norm and frank t-conorm. For aggregating the collection of information into a singleton set, we simplified the model of Cir-BCFFPA, Cir-BCFFPWA, Cir-BCFFPG, and Cir-BCFFPWG operators, and highlighted their valuable properties, called idempotency, monotonicity, and boundedness. For evaluating the above problem of energy-renewable resources, we constructed the technique of EDAS method under the presence of the Cir-BCFNs. In the end, we arranged the comparison between proposed and existing techniques based on some numerical examples to describe the validity and proficiency of the initiated information.

### 8.1 Limitations

The model of Cir-BCFS is very reliable, but if an expert provides the value of positive, negative, and radius functions in the form of the collection of the fuzzy values, then the proposed technique has failed, because of limited features. For this, we suggest constructing the technique of circular bipolar complex hesitant fuzzy sets which is suitable for depicting such kinds of problems.

### 8.2 Future directions

In the future, we will evaluate the novel technique of 3,4-quasirung fuzzy sets [35], quasi-rung orthopair fuzzy sets [36], Dombi operators based on confidence level [37], picture fuzzy payoff with application to cyberterrorism attacks [38] and applications in algebras [39,40]. The utilization of real-world applications is artificial intelligence, machine learning, game theory, data science, and decision-making to enhance the worth of the proposed theory.

## Funding

This paper is supported by the NRPU-HEC Pakistan Project Number 14662 and the joint project PSF (PSF-NSFC/JSEP/ENG/AJKUKAJK/01)-NSFC (12211540710).

## Data availability statement

No data was used for the research described in the article.

## CRediT authorship contribution statement

**Zeeshan Ali:** Writing – original draft, Conceptualization. **Khumara Ashraf:** Supervision, Investigation. **Khizar Hayat:** Validation, Conceptualization.

## Declaration of competing interest

The authors declare that they have no known competing financial interests or personal relationships that could have appeared to influence the work reported in this paper.
